# Phylogenomic insights into the polyphyletic nature of Altai falcons within eastern sakers (*Falco cherrug*) and the origins of gyrfalcons (*Falco rusticolus*)

**DOI:** 10.1038/s41598-023-44534-4

**Published:** 2023-10-18

**Authors:** Liudmila Zinevich, Mátyás Prommer, Levente Laczkó, Daria Rozhkova, Alexander Sorokin, Igor Karyakin, János Bagyura, Tamás Cserkész, Gábor Sramkó

**Affiliations:** 1 Koltzov Institute of Developmental Biology Russian Academy of Sciences, Moscow, Russian Federation; 2 All-Russian Research Institute for Environmental Protection, Moscow, Russian Federation; 3 University of Florida, Gainesville, FL USA; 4 HUN-REN–UD Conservation Biology Research Group, Egyetem tér 1, Debrecen, 4032 Hungary; 5 Evolutionary Genomics Research Group, Department of Botany, University of Debrecen, Egyetem tér 1, Debrecen, 4032 Hungary; 6 Sibecocenter LLC, Novosibirsk, Russian Federation; 7 MME – BirdLife Hungary, Költő utca 21, Budapest, 1121 Hungary; 8 Hungarian Natural History Museum, Baross utca 13, Budapest, 1088 Hungary

**Keywords:** Evolutionary genetics, Phylogenetics, Zoology

## Abstract

The Altai falcon from Central Asia always attracted the attention of humans. Long considered a totemic bird in its native area, modern falconers still much appreciated this large-bodied and mighty bird of prey due to its rarity and unique look. The peculiar body characteristics halfway between the saker falcon (*Falco cherrug*) and the gyrfalcon (*F. rusticolus*) triggered debates about its contentious taxonomy. The weak phylogenetic signal associated with traditional genetic methods could not resolve this uncertainty. Here, we address the controversial evolutionary origin of Altai falcons by means of a genome-wide approach, Restriction-site Associated DNA sequencing, using sympatric eastern sakers falcons, allopatric western saker falcons and gyrfalcons as outgroup. This approach provided an unprecedented insight into the phylogenetic relationships of the studied populations by delivering 17,095 unlinked SNPs shedding light on the polyphyletic nature of Altai falcons within eastern sakers. Thus we concluded that the former must correspond to a low taxonomic rank, probably an ecotype or form of the latter. Also, we found that eastern sakers are paraphyletic without gyrfalcons, thus, these latter birds are best regarded as the direct sister lineage of the eastern sakers. This evolutionary relationship, corroborated also by re-analyzing the dataset with the inclusion of outgroup samples (*F. biarmicus* and *F. peregrinus*), put eastern sakers into a new light as the potential ancestral genetic source of high latitude and altitude adaptation in descendent populations. Finally, conservation genomic values hint at the stable genetic background of the studied saker populations.

## Introduction

Large falcon species of the genus *Falco* Linnaeus (Falconidae, Falconiformes) are the most important birds used for falconry^1^. Amongst them, two species from the subgenus *Hierofalco* Kleinschmidt, 1901, the Holarctic gyrfalcon (*Falco rusticolus* Linnaeus, 1758) and, especially, the Palearctic saker falcon (*F. cherrug* Gray, 1834)—the two largest falcon species of the world—have been the most preferred ones^2^. These species represent the crown-clade of the monophyletic group of Hierofalcons^2–4^, which include the species *F. rusticolus*, *F. cherrug*, *F. biarmicus* (Temminck, 1834), *F. jugger* (Gray, 1834) and *F. subniger* (Gray, 1843), while saker falcons and gyrfalcons are currently considered as sister species^2–5^. The typical habitats of these two species are remarkably similar, as both of them have adapted to short-grass open environments: tundra and steppe, respectively. Nevertheless, there is also a stark contrast in distribution and ecological preferences between the two species: sakers live in arid grasslands across the Eurasian Steppe Zone^6^, from the Pannonian Basin through Asia Minor and Middle Asia to Mongolia and the Qinghai–Tibetan Plateau^7^, while gyrfalcons have a circumpolar distribution^8^.

For many centuries, harvest for falconry did not affect significantly populations of these species due to relatively low pressure and sustainable traditional practices^1^. By the twentieth century, however, habitat loss, direct persecution, pesticides, electrocutions, and unsustainable harvest have resulted in rapid global decline of sakers^7^, which was therefore listed as “*Endangered*” by International Union for Conservation of Nature (IUCN) Red List^9^. In contrast, the gyrfalcon is listed as *“Least concern”* by IUCN^10^, since its breeding population is quite stable in terms of occupancy and productivity^11^.

**Figure 1:**
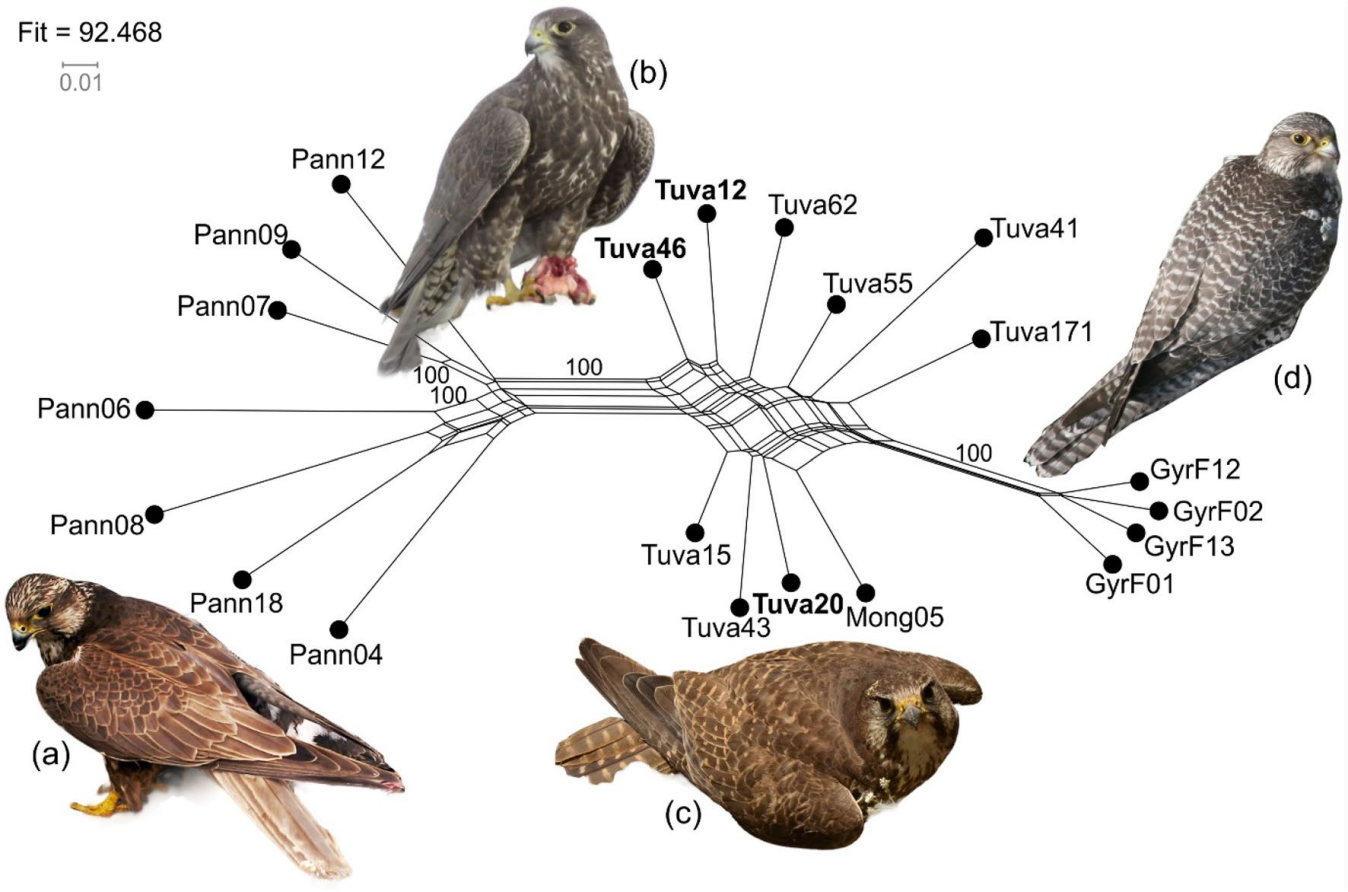
Genetic relationship between the studied saker falcon and gyrfalcon samples as depicted by a NeighbourNet network based on Kimura-2p genetic distance with non-parametric bootstrap (bs) support values built in SplitsTree. The numbers represent statistically significantly supported (bs > 75%) splits. Sample names follow Table 1. Samples of the ‘Altai falcon’ are indicated in boldface. Pictures of adult females of the studied taxa: (**a**) *Falco cherrug cherrug* (photo: János Bagyura), (**b**) Altai falcon (photo: Shane McPherson), (**c**) *Falco cherrug milvipes* (photo: Gábor Sramkó), (**d**) *Falco rusticolus* (photo: Alexander Sorokin).

The variability of gyrfalcons is clearly recognizable through the different morphs defined by the melanocortin-1 receptor gene^12^, but saker falcon also displays significant phenotypic diversity^13^. Falconers have long valued a large-bodied and dark morph (Fig. 1), called the ‘*Altai falcon*’^1^, which is endemic to the Altai-Sayan region at the border of the Russian Federation, Mongolia, China and Kazakhstan^13–15^. The unusual characteristics of this mighty bird gained a special reputation in early medieval culture as the totemic animal of Eurasian steppe nations such as ancient Magyars (Hungarians), who identified this bird with their mythological ancestor and called it ‘Turul’^16^. The Altai falcon has the highest frequency of occurrences in the Altai-Sayan Region of the Tuva Republic in the Russian Federation^17^. The rarity of this bird and the remoteness of the area it is found contributed to sparking debates about its systematic position.

Originally, the Altai falcon was described and named as two distinct species of the *Hierofalcon* group: *Falco [Hierofalcon] altaicus* Menzbier, 1891 and *Falco [Hierofalcon] lorenzi* Sushkin, 1901. Later, Sushkin^18^ recognized them as the same and phenotypically variable taxon with plumage ranging from normal saker coloration (see Karyakin^13^) to dark forms even within the same nest. Dementiev^19^ originally associated the dark form with gyrfalcon, but later revised his views^20^ and considered it to be a distinct subspecies. Additionally, other taxonomic accounts postulated the hybrid origin of these birds resulting from introgression from gyrfalcons^14,21–24^. To sum up, the taxonomic placement of the Altai falcon is highly contentious (reviewed by Karyakin^13^), being regarded either as (1) a morph or subspecies of the saker falcon, (2) a subspecies of the gyrfalcon, and (3) a hybrid between these two species. Nota bene, these views are not mutually exclusive and some authors changed their opinion through time.

However, the question of Altai falcon has not been addressed by using molecular genetic methods that provide enough resolution power^25^. Lack of phylogenetic resolution provided by traditional markers (i.e., mitochondrial DNA, microsatellites) is an issue in molecular systematics of *Hierofalcon*^3,4,26,27^, although genome-wide methods have delivered an unforeseen insight into saker phylogeography^28,29^. Currently, there are two main clinal population groups, connected only by occasional gene flow along a genetic gradient, recognized within sakers: a western and an eastern group^26^ resulting from an eastward diversification process^28^. As for the exact timing of this split, two radically different estimates exist. Pan et al., for which the species originated 34 kyr (1000 years) ago, set the split at around 2 kyr ago^29^. In more recent work based on whole-genome resequencing, Hu et al.^30^ reinterpreted previous results and assessed the separation of gyrfalcons and sakers to much earlier, *ca* 300 kyr ago, and the subsequent expansion of sakers from Europe to Asia at 41 (38–78) kyr ago. Morover, a recently published study^31^ set the divergence between the above-mentioned species at *ca* 109 kyr ago.

In spite of our basic understanding of the evolutionary history of the saker falcon, the origin Altai falcon has not been addressed in any genomic investigations even though it is on the brink of extinction in the wild^32^ mostly due to illegal harvest for falconry^1^. Consequently, there is a need for investigating the genetic status and elucidate the phylogenetic position of the Altai falcon using a genomic approach in order to set up conservation priorities and strategies^33^. Here, we apply such a technique, the reduced-representation genomic method, Restriction-Site Associated DNA-sequencing (RADseq)^34,35^, to gain insight into the phylogenomic placement of the Altai falcon by comparing birds with typical ‘Altai appearance’ to sakers from the eastern and western part of the range, and to their sister species, the gyrfalcon^2,6–8^. Here, we use the power of this genomic approach to test the possible hybridization pattern between the study groups, thus evaluating the likelihood of the hybrid origin invoked for the eastern saker.

## Results

### Genomic data generation

Our RADseq library sequencing of 21 samples of *Falco* samples (Table 1) yielded 159,284,527 raw, 150 bp long paired-end reads, of which 108,148,329 were retained after quality filtering and adapter trimming, leading to 4,180,573 ± 801,501 reads per sample on average (± standard deviation). Overall, 92% of the reads could be successfully mapped onto the saker reference genome (see Methods) that yielded 598,604 genotyped loci with 327,080 variant sites of which 136,655 were unlinked. The average coverage of the loci was 32 × (minimum 20.6 × , maximum 51.1 ×) with a mean number of sites per locus 311.

**Table 1:** Samples included in the study.

Sample name	Species	Country, region or settlement	GenBank accession no	Specific phenotype
Pann04	*Falco cherrug*	Hu: Enying	–	
Pann06	*Falco cherrug*	Hu: Gyömrő	–	
Pann07	*Falco cherrug*	Hu: Hegyeshalom	–	
Pann08	*Falco cherrug*	Hu: Hort	–	
Pann09	*Falco cherrug*	Hu: Karcag	–	
Pann12	*Falco cherrug*	Hu: Mezőtúr	–	
Pann18	*Falco cherrug*	Hu: Vadosfa	–	
Tuva12	*Falco cherrug*	Ru: Altai Republic	–	Altai form
Tuva15	*Falco cherrug*	Ru: Tuva Republic	OM937746	
Tuva171	*Falco cherrug*	Ru: Tuva Republic	OM937753	
Tuva20	*Falco cherrug*	Ru: Tuva Republic	OM937747	Altai form
Tuva41	*Falco cherrug*	Ru: Tuva Republic	OM937748	
Tuva43	*Falco cherrug*	Ru: Tuva Republic	OM937748	
Tuva46	*Falco cherrug*	Ru: Altai Republic	OM937750	Altai form
Tuva55	*Falco cherrug*	Ru: Tuva Republic	OM937751	
Tuva62	*Falco cherrug*	Ru: Tuva Republic	OM937752	
Mong05	*Falco cherrug*	Mongolia	–	
GyrF12	*Falco rusticolus*	Ru: Kamchatka-Chukotka	–	
GyrF13	*Falco rusticolus*	Ru: Kamchatka-Chukotka	OM937745	
GyrF01	*Falco rusticolus*	Ru: Kamchatka-Chukotka	OM937743	
GyrF02	*Falco rusticolus*	Ru: Kamchatka-Chukotka	OM937744	

Exact locations are not given due to conservation consideration. Samples sequenced for mitochondrial control region are indicated by the sequence’s GenBank accession numbers.

Country codes: *Hu* Hungary, *Ru* Russian Federation.

After filtering for missingness, the frequency of minor alleles, and Hardy–Weinberg-equilibrium (HWE), the final single nucleotide polymorphism (SNP) dataset consisted of 63,566 variable sites (dataset: “ref”), whereas the randomly exported sites from each locus were 17,095 (dataset: “rand”). The quality check of samples in the ref SNP dataset showed that all samples had mean and median genotype quality (GQ) score of 40 (i.e., the maximum value it can take up) and a mean read depth of 30.2 × (minimum 19.5, maximum 46.7), which corresponded to mean median value of 28.4 × (minimum 18, maximum 46) (Supporting Information).

As our phylogenetic results questioned the usage of gyrfalcon as an adequate outgroup, we decided to use the whole *F. biarmicus* and *F. peregrinus* genomes (see Methods for details) as a secondary outgroup^2,3,36^. Our bioinformatic mining of homologous regions identified 423 genomic parts that were unambiguously aligned to our RADseq loci in total length of 165,640 bp (mean ± s.d. = 391 bp ± 111 bp) with a minimum length of 157 bp and maximum length of 842 bp. In terms of phylogenetic information content, the dataset contained 1550 variable positions out of which 782 were parsimony-informative.

### Phylogenomic analyses

In the first place, we built a phylogenetic network using the Neighbour-Net algorithm based on Kimura-2p distance (Fig. 1). Three groups were clearly identified: (1) Pannonian sakers (i.e., western sakers), (2) Tuvan and Mongolian sakers (i.e., the eastern sakers), and (3) gyrfalcons from Far Eastern Russia. The Pannonian group and the gyrfalcons are approximately equidistant from the central Tuvan group and separated by 100% non-parametric bootstrap (bs) support. All Altai saker samples clustered homogeneously into the Tuvan group where they do not segregate into a subgroup in a statistically significant way. The fit-value of the network was 92.468, indicating the high probability for a tree to be fit to the data^37^.

**Figure 2:**
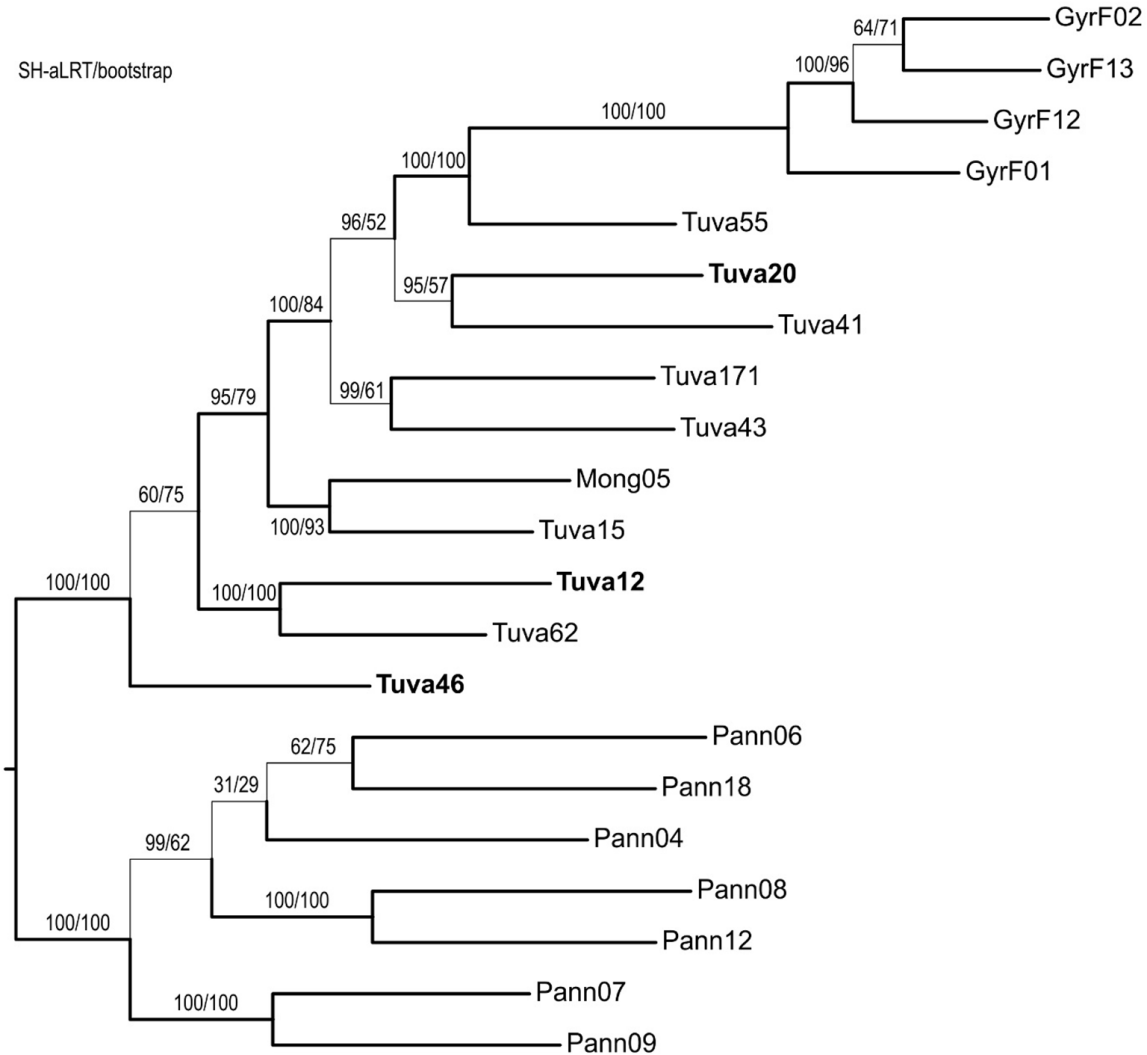
Phylogenetic tree based on 63,566 SNPs as reconstructed by IQtree using ascertainment bias in a maximum likelihood search. Support values (resulting from SH-aLRT and non-parametric bootstrap) are shown above the corresponding branch. Branches that receive statistical support (aLRT ≥ 80 and bs ≥ 75) are indicated by thick lines. Sample names follow Table 1. Samples of the Altai falcon are indicated in boldface.

Our phylogenetic tree reconstruction using a maximum-likelihood (ML) approach with an automated model selection taking ascertainment bias (ASC) into account yielded a well-resolved tree (Fig. 2). Rooting the tree with the European population^29^ yielded an unexpected topology, where—with full statistical branch robustness separating the populations as defined above—the Pannonian population is sister to the Tuvan population (including samples from Tuva and Mongolia), which leads to the gyrfalcons (i.e., the latter are a monophyletic daughter clade of the Tuvan birds that form a grade). Our analysis based on minimal ancestor deviation^38^ placed the root at the same place (i.e., between the Pannonian and the Tuvan populations) (Supplementary Fig. 4).

**Figure 3:**
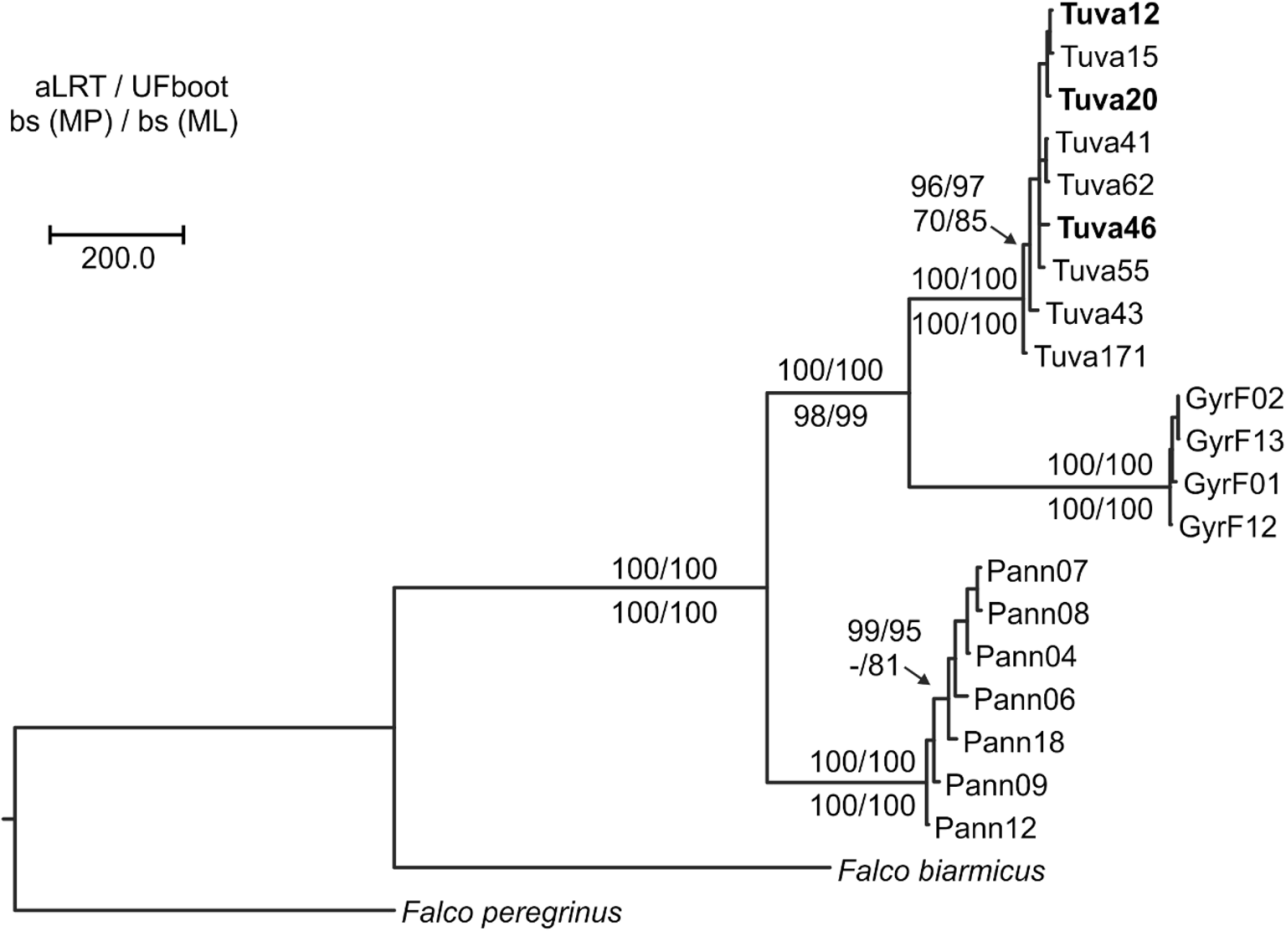
Phylogenetic tree based on phylogenetic analysis of concatenated data of 431 RAD loci with the inclusion of two outgroup samples, the lanner falcon (*Falco biarmicus*) as close, and the peregrine falcon (*F. peregrinus*) as distant outgroup species. The phylogenetic tree, displayed as a phylogram, is a representative one reconstructed by a maximum parsimony (MP) search that yielded 36 equally parsimonious trees. Topological robustness is indicated by displaying the branch support values coming from 1000 non-parametric bootstrap (bs) pseudo-replicates at the corresponding branches. The same data were used in a ML search that yielded a most likely phylogenetic tree with identical topology to the above shown one. Therefore, only support values resulting from SH-aLRT, UFboot and bs of the ML analysis are shown above at the corresponding branches. Branches that received support below 50 are not captioned. Sample names follow Table 1. Samples of the Altai falcon are indicated in boldface. The scale bar represents 200 mutation steps in the MP analysis.

As the placement of the root is highly important, we conducted an additional phylogenetic tree reconstruction with the inclusion of two formal outgroups (i.e., a taxon that has diverged earlier then the last common ancestor of the ingroup), *F. biarmicus* as a close relative from the *Hierofalco* group and *F. peregrinus* as a distant relative from the sister group of hierofalcons^2,3,36^. The maximum likelihood and maximum parsimony analyses yielded identical topology, where the root was placed between the Pannonian and the Tuvan populations (Fig. 3). This topology and the topology based on the “ref” dataset (Fig. 2) were also similar: there are only minor differences that receive no statistical support.

The phylogenetic placement of our ingroup samples is not fully resolved in our ML tree based on the “ref” dataset (Fig. 2). Although only some branches are supported statistically within the above populations (i.e., receive aLRT ≥ 85% and bs ≥ 75), signals of structure within the Tuvan population can be seen. More importantly, Altai birds are scattered along the tree within the grade (i.e., the paraphyletic group) of the Tuvan population, and the sample Tuva20 is separated by significantly supported branches from the other two such samples. Additionally, Altai sample Tuva12 also forms a fully supported clade with the non-Altai sample Tuva62. Therefore, it implies the non-monophyly of the Altai falcon samples within the Tuvan grade, and the paraphyletic nature of eastern sakers without Far Eastern gyrfalcons.

### Population genomic analyses

**Figure 4:**
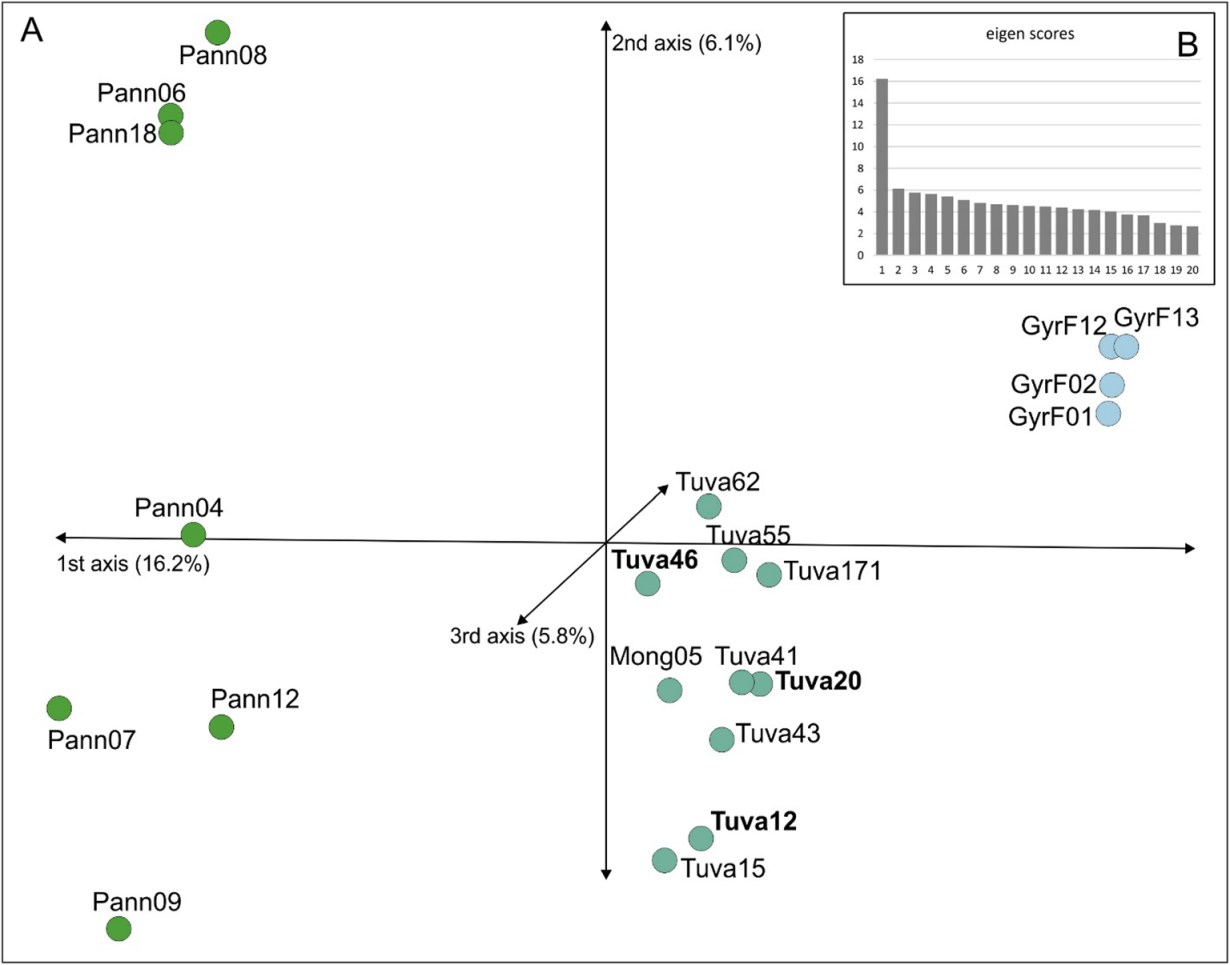
Genetic relationship between the studied saker individuals based on 17,095 unlinked SNPs as depicted by a Principal Component Analysis of allele frequencies (**A**). The first axis account for 16.2%, whereas the second axis 6.1% of total variation (see also the insert **B**). Sample names follow Table 1. Samples of the Altai falcon are indicated in boldface.

We explored the “rand” dataset, which only contains unlinked SNPs, using ordination and k-means clustering approaches. The first three axes of the Principal Component Analysis (PCA) of SNP frequencies (Fig. 4) accounted for 28.1% of the total variation (Fig. 4B). The samples mostly separated (Fig. 4A) along the first axis (explaining 16.2% of the total variance) into three groups equidistant from each other: (1) the Pannonian samples, (2) the Tuvan samples including the Mongolian samples, (3) the gyrfalcon samples. The Altai falcon samples were dispersed in the Tuvan group.

**Figure 5:**
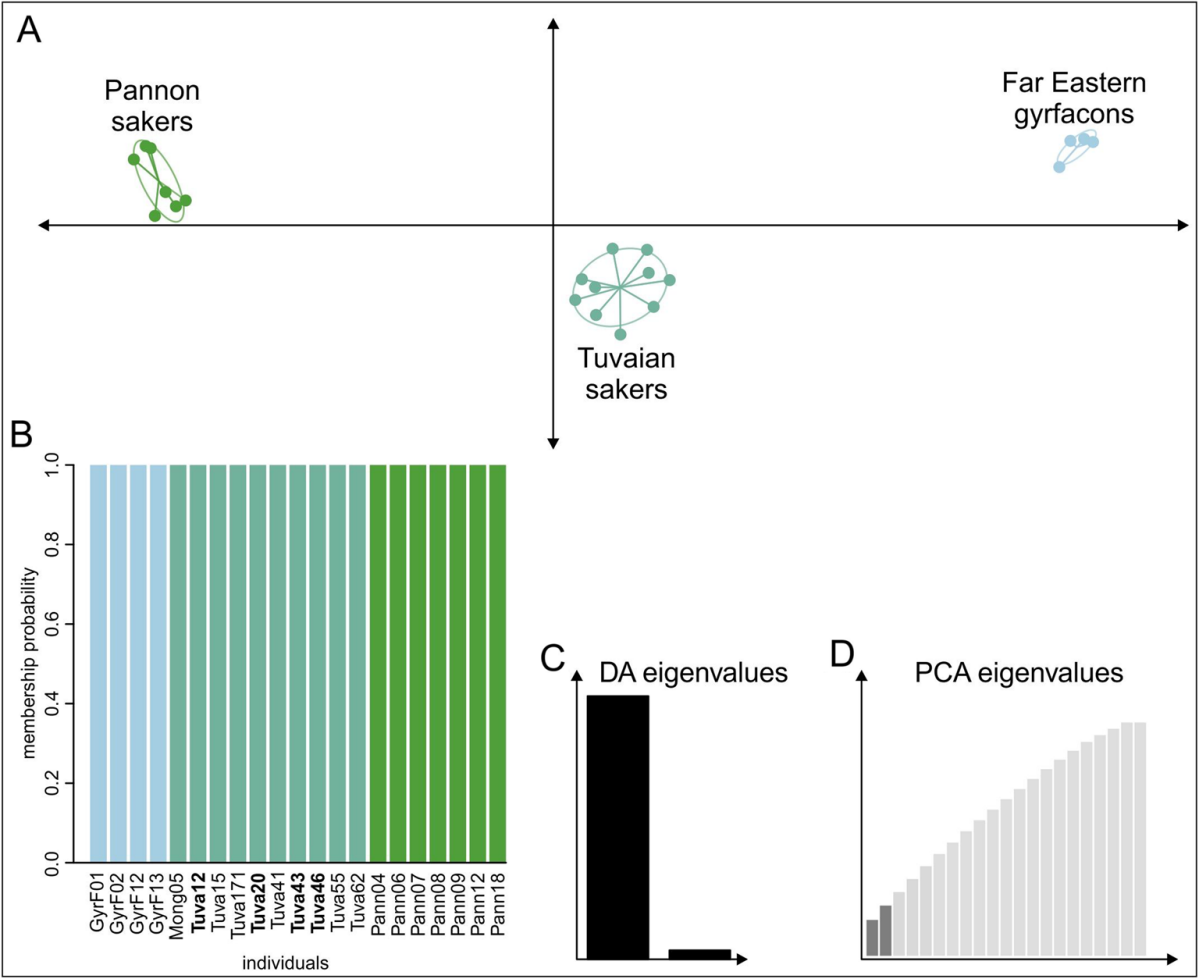
Genetic relationship between the studied saker individuals based on 17,095 unlinked SNPs as depicted by discriminant analysis of principal components (DAPC). The scatterplot of samples represented as dots and inferred groups as inertia ellipses (**A**). Individual group membership probabilities of DAPC indicating clear grouping of individuals into the three DAPC group (**B**) with samples of the Altai falcon indicated in boldface. Eigenvalues of the underpinning linear discriminant analysis (**C**) and the principal component analysis (**D**) are also displayed.

A multivariate method, Discriminant Analysis of Principal Components (DAPC), was used to infer groups within the samples. DAPC analysis found K = 3 as the optimal number of clusters for our genomic data, and separated the samples into three, highly distinct groups (Fig. 5): (1) Pannonian sakers, (2) Tuvan sakers, and (3) gyrfalcons. The inclusion of individuals in each group was highly certain with almost 100% membership probability (Fig. 5B) and all the Altai sakers were included in the Tuvan group with 1.0 membership probability.

**Figure 6:**
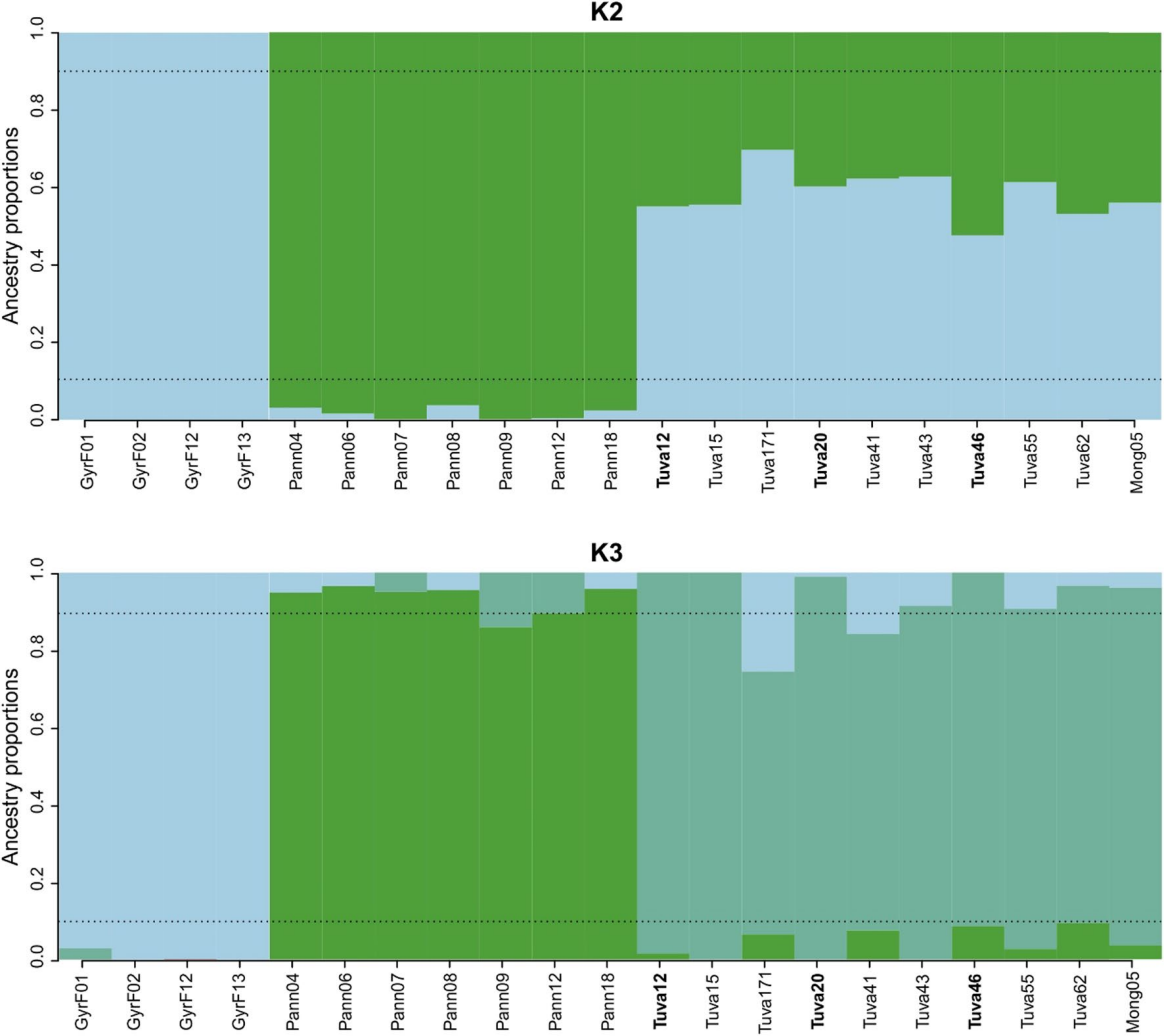
Co-ancestry-based sparse non-negative matrix factorization (snmf) analysis of 17,095 unlinked SNP data of the studied falcon samples depicted as ancestry proportion barplots when assuming two (K2) or three (K3) ancestral populations. The threshold of upper and lower 10% ancestry proportion is indicated by a dashed line. Sample names follow Table 1. Samples of the Altai falcon are indicated in boldface.

We also accomplished a co-ancestry-based sparse non-negative matrix factorization (snmf) analysis of SNP data to explore potential admixture between the samples (Fig. 6). K = 2 was suggested as the optimal number of clusters by the cross-validation algorithm of the snmf function, but K = 3 had a little higher entropy value indicating the next possible probability (Supplementary Fig. 5). At K = 2, gyrfalcons and Pannonian sakers showed no significant admixture; at the same time, the Tuvan population showed large-scale admixture with both of them at K = 2. At the next hierarchical level of grouping at K = 3, the three populations (i.e., Pannonian sakers, Tuvan sakers, gyrfalcons) clearly separated into three groups without much admixture. Significant admixture was observable in a few individuals with critical values: there is admixture above the 10% threshold between the Tuvan and the gyrfalcon population (see samples Tuva171 and Tuva41) and between the Pannonian and Tuvan sakers (see sample Pann09). All Altai samples showed the same admixture pattern as the Tuvan sakers.

Genetic differentiation between the studied populations using Weir and Cockerham weighted F_st_ estimate showed that gyrfalcons are the most genetically differentiated from the two saker populations (gyrfalcon *vs*. Pannonian saker F_st_ = 0.21; gyrfalcon *vs*. Tuvan saker F_st_ = 0.09), and the lowest level of differentiation (F_st_ = 0.06) can be seen between the two studied saker populations. The largest genetic differentiation—as measured by mean F_st_ of the populations—occurred in gyrfalcons (mean F_st_ = 0.1), followed by Pannonian (mean F_st_ = 0.09) and Tuvan sakers (mean F_st_ = 0.05).

**Figure 7:**
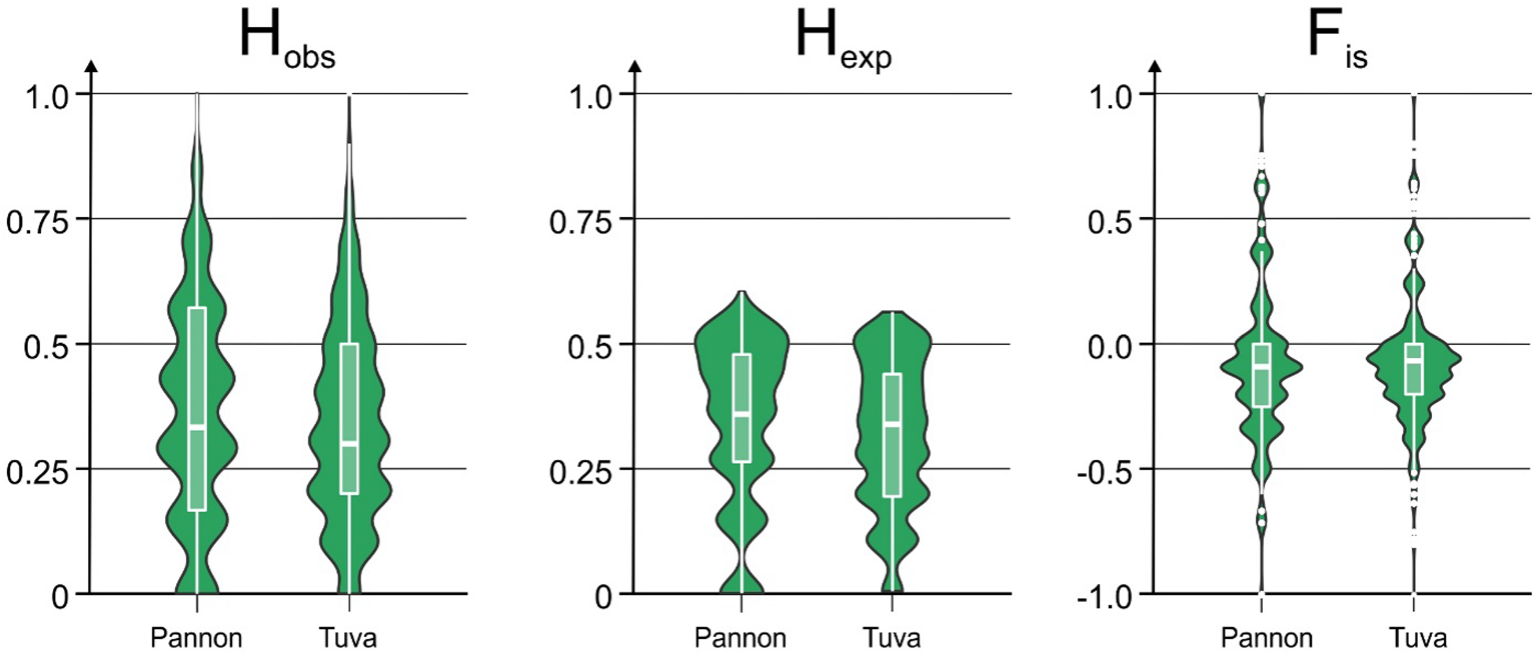
Basic population genomic values (H_obs_—observed heterozygosity, H_exp_—expected heterozygozity, F_is_—inbreeding coefficient) based on 17,095 unlinked SNPs depicted by violin plots for the two studied saker falcon (*Falco cherrug*) populations.

Finally, we were interested in the basic conservation genomic characteristics of the endangered Hungarian and Tuvan populations, so we calculated genome-wide heterozygosity and inbreeding values. In all cases the Hungarian population displayed slightly higher mean and median values for the analyzed genetic diversity values (Fig. 7).

### Mitochondrial data analyses

**Figure 8:**
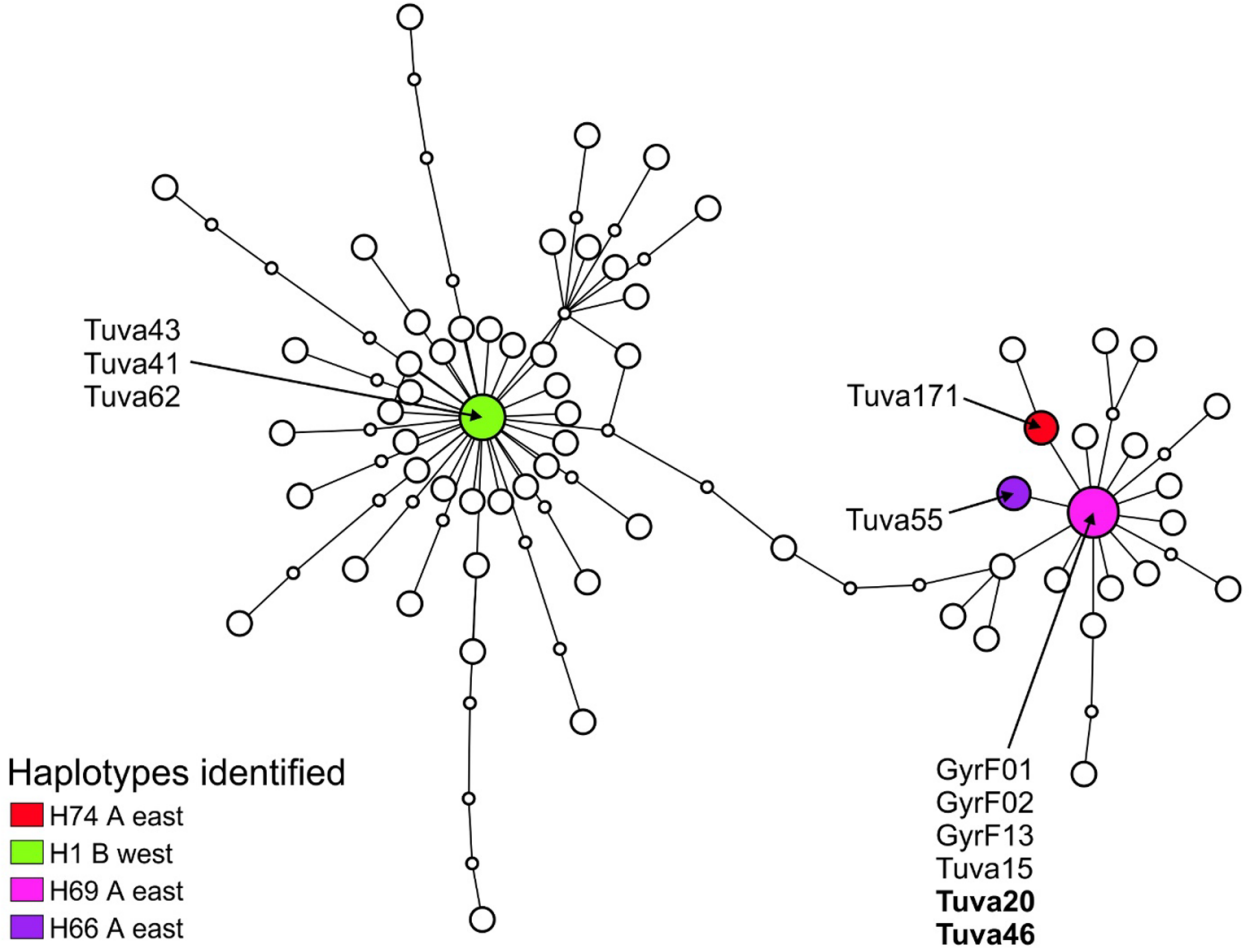
Parsimony-based clustering of mitochondrial control region sequences of sakers from the eastern population and a gyrfalcon using the software TCS. The mitochondrial dataset of Nittinger et al.^26^ is used for the analysis and their samples are unlabeled above. The placement of individuals which are genotyped in both this and our RADseq analysis are highlighted. Sample names follow Table 1. Samples of the Altai falcon are indicated in boldface.

In order to place our results into a comparative context, we sequenced the mitochondrial (mt) control region (i.e., the D-loop) used by Nittinger et al.^26^ in their comprehensive work. We focused only on the eastern sakers as it is already known that their western counterparts only yield haplotypes from the western (‘B’) haplogroup^26^. We successfully sequenced a 415 bp control region fragment and aligned it with the homologue sequences published by Nittinger et al^26^ (GenBank accession numbers: OM937743–OM937753). Haplotype building based on statistical parsimony could connect the network with 95% “parsimony probability” at eight steps and resulted in a haplotype network identical to that one published before^26^ (Fig. 8). Our eastern saker samples either fell into the western (samples Tuva41, Tuva43, Tuva62 holding haplotype H1) or the eastern (Tuva15, Tuva20, Tuva46 holding haplotype H69; Tuva55 holding haplotype H66; Tuva171 holding haplotype H74) haplogroup. Samples Tuva12 and Mong05 were not included in this analysis. Gyrfalcon samples GyrF01, GyrF02 and GyrF13 were included in the eastern group as it possessed the central haplotype of this group, haplotype H69. Altai birds all yield the same central haplotype (H1) of the eastern group.

### Introgression analyses

**Figure 9:**
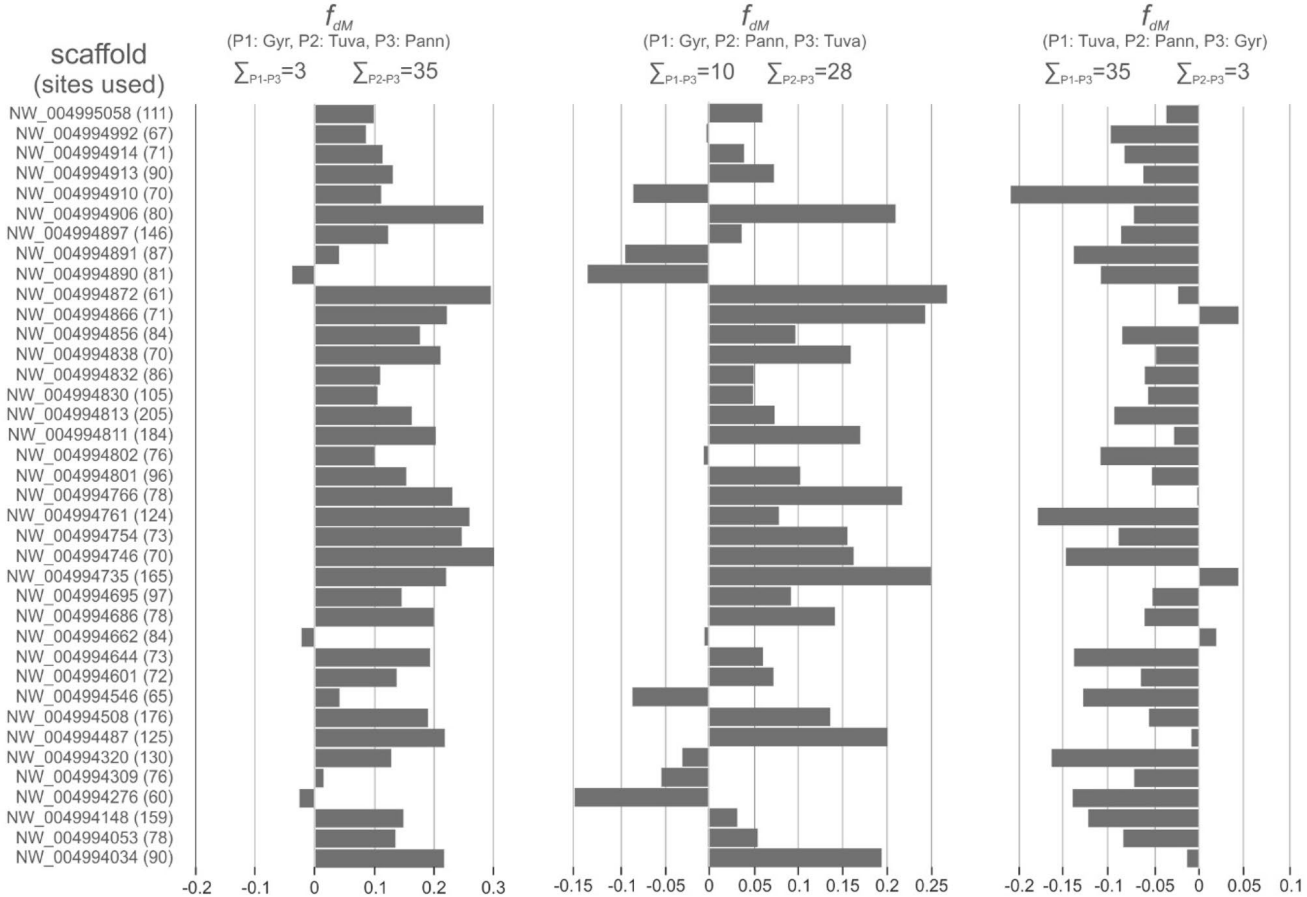
Statistics of introgression along the sampled part of the saker genome expressed by the *F*_*dM*_ value of each scaffold used for the analysis. The statistics is positive if there is gene flow between population 2 (P2) and population 3 (P3), and negative if the gene flow exists between population 1 (P1) and P3. The number of sites used by the test in each scaffold is indicated in parentheses.

A recent paper by Hu et al.^30^ was published during the review process of this work that prompted us to conduct exact statistical tests to assess the proportion of derived alleles between the clades identified (see Fig. 2) in an ABBA/BABA test. This test checks the distribution of shared alleles between three populations in the presence of an outgroup that represents the ancestral allele. Three tests were done: (1) we ran an ABBA/BABA test with *F. biarmicus* used as outgroup in the CalcPopD function. The test of an ABBA/BABA pattern (Patterson’s D-statistics) with 100 jackknife resampling of block size of 850 bp (which was set to be higher than the longest RAD locus to eliminate potential linkage) proved to be significantly positive: D (gyr,tuva,pann,biar) = 0.232 (SD = 9.8 × 10^–3^) (Z-score = 23.57) (*p* = 0.000), thus indicating introgression and gene flow between the studied populations. To further explore this relationship, we ran two additional analyses: (2) sliding windows analysis of *F*_*dM*_ statistics^39^ (Fig. 9.) and (3) the formal test of admixture for three populations (*f*_*3*_-test^40^). As the pseudo-haploid genome we used as outgroup can be suboptimal due to the lack of complete allelic variation information, we decided to focus on the latter two statistics which describes the relationship between the three ingroup populations and does not require a fourth (outgroup) sample.

In the case of the *F*_*dM*_-statistics, it is positive if there is gene flow between population 2 (P2) and population 3 (P3), and negative if the gene flow exists between population 1 (P1) and P3. Our results clearly indicated gene flow between eastern and western sakers with 28 and 35 RAD regions showing introgression from western to eastern sakers and contrariwise, respectively. At the same time, 35 and 10 genomic regions indicated gene flow from the eastern sakers to gyrfalcons, and vice versa, respectively. The potentially introgressed number of loci between gyrfalcons and western sakers (3) is the lowest (Fig. 9).

Finally, we calculated the *f*_*3*_-statistics, which tests if a target population is the result of mixture from two source populations. Significant negative value can only be found if we assume a set up of the experiment by assuming Pannonian sakers and gyrfalcons as source populations, and Tuvan sakers as target population (*f*_*3*_ value = − 0.046762; Z-score = − 27.507). The other population arrangements were all positive (i.e., indicative of no admixture; Supplementary Table 1). Thus, allele frequencies of Tuvan population were intermediate between Pannonian sakers and gyrfalcons, consequently eastern sakers have admixed origin but not necessarily with these source populations (see below).

## Discussion

### Phylogenetic and phylogeographic patterns

The employment of a genome-wide method based on thousands of SNPs delivered a detailed insight into the phylogenetic relationships among our targeted populations, where large geographic groups were clearly separated. This is in accordance with previous works finding a strong phylogeographic structure within sakers consisting of an eastern and a western main group^26,29,41^. The most surprising result in our analyses is the similar genetic distance of eastern sakers with gyrfalcons and western sakers (Figs. 1, 2, 3, 4, 5 and 6). This was quite unexpected in the light of phylogenetic tree reconstruction results showing gyrfalcons as a monophyletic unit within eastern sakers (Figs. 2, 3), with the latter being therefore paraphyletic without the gyrfalcons. This result is unexpected as we originally included gyrfalcon samples as outgroup. Although the interpretation of our phylogenetic tree strongly depends on rooting, the inclusion of a distant (*F. peregrinus*) and a close (*F. biarmicus*) outgroup sample (Fig. 3), the rooting based on minimal ancestor deviation analysis (Supplementary Fig. 4) along with previous transcriptomic data of eastern and western sakers^29^ leave no doubt that western sakers are the basal clade within *F. cherrug*. This interpretation is further corroborated by the well-established crown position of the species-pair *F. cherrug* and *F. rusticolus* in various phylogenetic analyses^2–5^. The minor discrepancies between our phylogenetic trees (Figs. 2 and 3) may result from the reduced information content of the two datasets; the “ref” dataset contains two orders of magnitude more data. Above all, we are convinced that the right position of the root—as also indicated by Pan et al.^29^—is correctly placed between the Pannonian and the Tuvan populations, which also implies that gyrfalcons are the sister group of eastern sakers (Fig. 3).

The prevailing hypothesis on the origin of the Hierofalco group was based on results of mt DNA analyses^2,3^, which suggested the Pleistocene origin of contemporary species through several waves of colonization of the Palearctic from Africa. Later, this hypothesis was further elaborated with the assumption of broad secondary hybridization based on nuclear microsatellite and mitochondrial variation^26^. Although nine microsatellite loci were reported to be useful in discerning gyrfalcons and sakers in a small number of samples^27^, our extensive experience—based on genotyping of *ca* 200 birds (exactly: 60 gyrfalcons and 148 sakers) with microsatellite panel including the same loci—does not confirm their species-specificity on a larger sample (unpublished results of Zinevich et al.). Similarly, an extensive sharing of mt control region haplotypes for different species is reported, which was interpreted as hybridization and/or incomplete lineage sorting^4,26,27,42^. In contrast, the phylogeographic analysis of gyrfalcons^42^ based on mitochondrial and nuclear DNA variation found a 1.5 k-long fragment of their mitogenome to be a private haplogroup originating from eastern sakers (provided that western sakers are ancestral) and characterized by a star-like topology and lower haplotypic diversity compared to sakers. These elements point to gyrfalcons as sister group of eastern sakers and not as the result of introgression that would lead to shared haplotypes between them. These mitochondrial data are consistent with our interpretation and results, although the unidirectional introgression put forth previously can also explain this pattern^30^. Having said that we still consider the direct ancestry of gyrfalcons from eastern sakers a biologically meaningful hypothesis based on the present results.

The reconstruction of ancestral population sizes based on transcriptome data suggests a recent Holocene diversification (*ca* 34 kyr) for the saker falcon and an eastward expansion^29^. In contrast, WGS data suggests an older split (*ca* 300 kyr) of ancestral sakers from gyrfalcons, yet, this is not supported by our findings. A third stem age of sakers was estimated by Al-Ajli et al.^31^ who report the separation at 109 kyr ago. Given this uncertainty in saker stem age, we cannot set the exact timing of evolutionary events, but hypothesize below the possible evolutionary history based on our results.

The latest evolutionary event in the history of sakers and gyrfalcons must have taken place during one of the colder periods of the Pleistocene in the Altai Mountains. Paleo-environmental reconstructions agree on the occurrence of large tundra-steppe grasslands in Eurasia^6^. Recent paleoecological reconstructions^43–46^ agree that most of the eastern European steppe belt, central and western Siberia hosted a mosaic tundra-steppe environment in which mixed birch-pine open woodlands were present as opposed to dense, closed forests. North-east Siberia, as currently understood^43^, had relatively high net primary production for mesophiolous herbs, which agrees with the presence of rich megafauna there^44^. This landscape south of the permafrost zone must have also been rich in rodents and could have been the ancient habitat of the saker. This could be the period when the species expanded its distribution range the most, as supported by effective population size reconstructions^29–31^. This large range became fragmented most likely in the Holocene, similar to that of the peregrine falcon (*F. peregrinus*)^47^. Most importantly, ancestral gyrfalcons may have originated from eastern sakers by forming of pre-zygotic, spatial and/or behavioral isolation. Part of ancestral eastern sakers better adapted to hunting large prey might have followed the retreat of the ice shield and have become isolated from that source area as a consequence of the northward shift of the tundra zone and partly the formation of the taiga zone, as already put forth by Potapova et al.^25^. This could have happened recently on an evolutionary timescale. Genetic differentiation (F_st_) values (see Results) between our studied populations further underpin this hypothesis as gyrfalcons show the highest level of differentiation (as measured by meanF_st_). This can be the result of a single, recent colonization event of the tundra habitat, which resulted in significant genetic differentiation due to founder effect, as already pointed out by Johnson et al^42^ based on the analysis of SSRs and mtDNA. The unbalanced genetic admixture between the gyrfalcons and eastern sakers (i.e., only from eastern sakers to gyrs and not *vica versa*) in our *F*_*dM*_ analysis (Fig. 9) further argue for the separation of the gyrfalcons as daughter species.

A closer inspection of introgression using the *F*_*dM*_-statistics (Fig. 9) revealed genetic exchange between western and eastern sakers which explains the significant D-statistics (see Results). The analysis of direction of admixture (Fig. 9) could not identify remarkable gene flow from gyrfalcons to eastern sakers, but indicated admixture in the opposite direction. This observation alone does not support the hypothesis of the hybrid origin of eastern sakers as the result of cross between western sakers and gyrfalcons. On the contrary, LEA analysis based on ancestry proportions found K = 2 as the most probable grouping (Supplementary Fig. 5), which shows the intermediacy of the eastern sakers (Fig. 6). Similarly, *f*_*3*_-statistics clearly indicated the significantly admixed nature of eastern sakers resulting apparently from gene flow from western sakers and gyrfalcons (Supplementary Table 1). This apparent contradiction may result from two sources: (1) the sensitivity of the analyses to phylogenetic structure: eastern sakers are intermediate between western sakers and gyrs in the genetic space (Figs. 4 and 5) as a result of very recent isolation and (2) the lack of an explicit outgroup population may also influence this result. Nevertheless, a statistically significant (i.e., Z-score < − 3) negative value (i.e., the test indicates admixture) can be misleading signal in case of the ‘outgroup case’^40^. If western sakers split off more anciently than gyrfalcons (Figs. 1, 2 and 3), *f*_3_-statistics with these source populations cannot show real phylogenetic history due to this phenomenon^40^. If we analyze the direction of admixture further (Fig. 9), it can come as no surprise that gene flow is unidirectional from eastern sakers to gyrfalcons. If the latter is the descendent of the former ancestral population, this sort of “gene flow” is the only possible genetic exchange as a result of daughter relationship of gyrfalcons. Taking all results into consideration, we hypothesize a recent evolutionary origin of *F. rusticolus* as suggested by the high number of shared alleles with eastern sakes with no or very limited gene flow between these two populations.

In light of this result, we may need to re-consider the finding of Hu et al.^30^ on the admixed nature of eastern sakers and gyrfalcons. If our phylogenetic hypothesis mirrors the correct evolutionary relationship between the studied populations, the shared genomic elements between gyrfalcons and eastern sakers can be explained by common ancestry. Indeed, if gyrfalcons isolated from the eastern sakers of the Altai Mountains, they could carry those genetic elements which could serve as pre-adaptations to tundra environments (and to the Qinghai–Tibet Plateau) and—along with other genomic regions—are now observed as shared between eastern sakers and gyrfalcons. This would also explain the significant admixture found in our study (Fig. 9) and repeatedly reported by others^30,31^. Nonetheless, this interpretation does not affect the significance of pre-adapted genetic parts reported by Hu et al. in the successful colonization of the Qinghai–Tibet Plateau by ancestral western Mongolian sakers.

The inclusion of the same samples in both in the nuclear RADseq-based analysis of admixture (Fig. 6) and the re-analysis of the mtDNA dataset of Nittinger et al.^26^ (Fig. 8) allow for a direct comparison of the placement of samples in the nuclear and mitochondrial genetic space. In this respect, the nuclear dataset shows a single case of a significant indication of admixture between the western and eastern sakers in case of individual Tuva171 (Fig. 6), which held haplotype H74 belonging to the eastern haplogroup (Fig. 8). Consequently, we could not identify a case of mito-nuclear discordance^48^. Additionally, our larger scale screening of control region haplotypes in Altai birds^32^ revealed the existence of haplotypes from the two main haplogroups in the studied samples. These observations, together with the *F*_*dM*_ statistics (Fig. 9), argue for gene flow between the two saker populations. However, this result does not influence the large-scale phylogeographic structure (Figs. 1, 2, 3, 4, 5 and 6) and makes gene flow to be rather occasional or a non-recent (historic) event that occurred in the evolutionary history of the species.

Indeed, contemporary long-distance dispersal between eastern and western sakers has not been confirmed by any other techniques so far. Even if roughly 5000 individuals were ringed across the entire range since 1950 (Prommer pers. comm.), and about 200 were satellite tracked since 2004^49–52^, not a single ring recovery or tracking result suggested gene flow between the eastern and western populations. Occasionally, juvenile saker falcons from Central Europe roam to east as far as European Russia and West Kazakhstan, but the tracked individuals always returned to the fledging region^50^. The furthest dispersal ever recorded was of approximately 1200 km and consisted of a female saker falcon tagged in West Romania (Pannonian region) that settled and bred in Crimea^53^. However, both locations are within the western saker population’s range. Some of the eastern saker falcons tagged in the Altai Mountains roamed west as far as western Kazakhstan^54^, but no evidence for further dispersal on that direction were found. The normal migration route of eastern sakers is on the north–south axis^49,55^. Finally, Pan et al.^29^ reported a 2–4 orders of magnitude higher eastwards migration rate than the opposite direction on an evolutionary timescale. This observation can also explain our finding of gene flow between saker populations, which is probably the evidence of historical event that occurred at a minimum extent. The now extinct^7^ populations of European Russia and those from Kazakhstan now on the brink of extinction likely mediated this gene flow. In conclusion, our current understanding of the large-scale movements of sakers does not support the hypothesis of contemporary genetic exchange between eastern and western sakers.

As an alternative to ongoing hybridization, we can refer to incomplete lineage sorting^56^ (ILS) as the source of eastern mtDNA haplotypes in the western area and vice versa. Although it is notoriously difficult to separate ILS from hybridization^57^, we are inclined to make ILS liable, at least partly, for the incongruent distribution of haplotypes between the two main saker groups. As the isolation of the eastern and western populations took place in the Holocene, at least according to one estimate^29^ (i.e., very recently on an evolutionary timescale), we may correctly assume that even fast-mutating mtDNA could not undergo complete lineage sorting during this time (see also^4,27,42^), thus, casting doubts about the existence of at least occasional contemporary hybridization between the two saker groups. This result clearly demonstrates why fast-evolving mitochondrial haplotypes alone cannot provide an ultimate insight into the evolutionary history of Hierofalcons.

Apart from the evident evolutionary historical relevance of the phylogenetic finding, it also raises a severe taxonomic question of whether gyrfalcons should be considered to be conspecific with sakers. Although a more thorough sampling is clearly needed in sakers and gyrfalcons to confidently address this question, our current preliminary look suggests that treating gyrfalcon as a saker subspecies (i.e., *F. cherrug rusticolus*) could be an apparently correct taxonomic treatment from genetic perspective as paraphyly alone cannot argue against the species-level separation of a young^42^ taxon according to the unified species concept^58^. Having said this, we ought to emphasize that further studies are needed to establish the firm taxonomy of the clade formed by *F. cherrug* and *F. rusticolus*, especially because such newly formed species may have other traits (e.g. ecological niche differences, behavioral differences) arguing for the species-level separation. Clearly, the limited admixture we detected between gyrfalcons and sakers (Fig. 9) let us argue for the higher level separation (i.e., at the species level) in spite of their sister group relationship.

Finally, we need to draw attention to the incomplete taxonomic sampling in our dataset and those discussed above; none of these studies included all taxa (i.e., all geographical populations of sakers and gyrfalcons) and explicit outgroups to provide a comprehensive picture. We need to acknowledge that the lack of any samples from the intermediate geographic regions (e.g. Crimea, West Kazakhstan) between the Altai and the Pannonian Basin (i.e., the peripheries of the distribution), could confound the phylogeographic picture if there is a clinal variation in genetic structure. Keeping this in mind, we need to echo that a more thorough understanding of the evolutionary relationships can only be obtained with a more complete taxonomic sampling^59^.

### The origin of the Altai falcon

Our present study attempts to clarify also the genetic background of the phenotypic variability of two large falcon species within the *Hierofalco* subgenus using a genome-wide approach, RADseq, that effectively and representatively samples the whole-genome^35,60^. Disregarding an early attempt by Sushkin in 1938^17^ to study the heritability of the morphological characteristics of an Altai male falcon, the origin and taxonomic status of these birds were not investigated by genetic methods. Here, we showed that Altai falcons are deeply nested within the genetic variability of eastern sakers (Figs. 1, 2, 3, 4, 5, 6, 7 and 8). In this respect, the most relevant result is our phylogenetic tree based on > 63 k SNPs (Fig. 2) which provides evidence on the non-monophyly of the samples showing an ‘Altai’ phenotype.

Nevertheless, the above result still leaves the question whether the non-monophyly of the Altai birds is either the result of hybridization between different saker subspecies (e.g. suggested by Karyakin^13^) or between the eastern saker and gyrfalcons (e.g. advocated by Ellis^14^, Potapov and Fox^21^, Potapova et al.^25^, Pfander^22,23^). This question was addressed by our estimate of admixture proportions via the snmf analysis as implemented in LEA (Fig. 6) and the test of admixture addressed by *f*_*3*_-statistics (Supplementary Table 1) and *F*_*dM*_-statistics (Fig. 9), which brought no evidence for admixture between any of the included samples of Altai birds. The exact test of introgression between the three clades we identified (see Fig. 2) showed the most remarkable genetic exchange between the eastern and the western sakers and only from eastern sakers to gyrfalcons in this respect (Fig. 9). The results of the *F*_*dM*_ statistics demonstrate the genetic exchange between the eastern sakers and both the western sakers and gyrfalcons. Similarly, the *f*_*3*_-statistics indicated the admixed nature of the Tuvan population, but not as a result of ancestry from Pannonian sakers and gyrfalcons (Supplementary Table 1). Consequently, these observations on admixture cannot exclude the hybrid origin of the eastern birds, and so the Altai form which is nested deeply in this clade (Figs. 1, 2, 3, 4, 5 and 6).

In this respect it is especially relevant to refer to two recent papers^30,31^ utilizing a genomic approach on the origin of eastern sakers which were published during the review process of our work. Hu et al.^30^ accepted the general view on gyrfalcons being the sister species of (a monophyletic eastern and western) saker, and thus used the former as outgroup in their analyses. Consequently, their test for introgression using the *f*_3_-statistics (their Supplementary Table 6.) reports very similar values to our results (Supplementary Table 1) and conclude on the existence of gene flow between gyrfalcons and eastern sakers. This interpretation only holds if we accept their phylogeny with ancestral western sakers splitting from ancestral gyrfalcons *ca* 300 kyr ago. Thus, we need to stress the different interpretation of these result between our research groups: while we regard this as a proof of an apparently admixed nature of the eastern sakers resulted from genetic intermediacy (Fig. 1) between western sakers and gyrfalcons, Hu et al.^30^ interpret the same result as a “strongly supported admixture between gyrfalcons and East sakers”.

The other study^31^, at the time of completing this work only available as a preprint, compared whole-genomes of captive sakers to field-collected western Mongolian sakers (either designated as “saker-like” or “gyrfalcon-like” in morphology) and to field-collected gyrfalcons. Based on this sampling, the authors found western Mongolian sakers to be admixed between gyrfalcons and captive sakers. Moreover, they report the “gyrfalcon-like” Mongolian sakers more admixed with gyrfalcons than those they classified as “saker-like” Mongolian sakers. We must note that the exact geographic origin of the captive sakers, which served as the basis of comparison between the different populations, is unknown and that makes any conclusion questionable. Also, if those birds represent the European population (i.e., western sakers), then the finding of the preprint is exactly mirroring what we reported here: the eastern sakers can be interpreted as the result of admixture with appropriate intermediate genetic position between western sakers and gyrfalcons.

We need to further reflect to a very important taxonomic conclusion: the authors argue for the species-level separation of the (gyrfalcon-like) western Mongolian sakers. We need to note the failure of clarifying the geographic origin of the “pure” sakers in that work. However, if they are representative of the western sakers, their results corroborate ours: gyrfalcons are the sister group of eastern sakers, and the intermediate genetic position of the latter can be the reason for finding a significant admixture between (western) sakers and gyrfalcons (see the preceding chapter for further discussion). Although in their phylogenomic analyses two mitogenomes of gyrfalcon-like sakers form a monophyletic unit, this does not hold for the W-chromosome haplotype of the three gyrfalcon-like saker specimens they examined. We regard the former result as non-conclusive (because only two samples show this pattern and there is no statistical support reported for this relationship), and the latter as a further support for our results: eastern sakers displaying an ‘Altai’ phenotype do not form a monophyletic unit, thus, they are with different phylogenetic origin. These results as now described in the above preprint do not support elevating eastern sakers with an ‘Altai’ phenotype to be at the species level.

In light of our genomic results, we consider the Altai birds to be phenotypic morph of eastern sakers, thus, echoing the opinion of Pfeffer^15^, who went further on to speculate that this form represents an atavistic, ‘ancestral’ form of sakers. Although we cannot exclude this possibility, in our view it is more likely that the Altai phenotype appears sporadically in the populations probably as a result of a point-like change (e.g. a structural mutation or a point mutation) in the genome. As mentioned earlier, the dark coloration of gyrfalcons is clearly associated to a single gene^12,61^, and this can also be behind the origin of the dark form in eastern sakers. However, several other genetic or epigenetic mechanisms can also stand behind this phenomenon. For instance, the insertion of a single transposable element can greatly alter the phenotype of birds as was recently demonstrated by Weissensteiner et al^62^ in the case of a songbird species. Whether this change in genotype is a result of an external stimulus (e.g. environmental conditions during the early embryonic life) or fully accidental remains a question. The geographically confined occurrence of this form (i.e., centered on the Altai-Sayan Mts.), which shows an increasing gradient towards the highest part of the mountains^17^, hint at the existence of such environmental influence (or influences) that triggers the appearance of the Altai form. As such alterations to phenotype during early embryonic life can be predictively or immediately adaptive^63^, it can explain the growing density of dark plumage phenotype at high altitudes where more harsh environmental conditions exist. If gyrfalcons are descendent of ancient eastern sakers as our results suggest (Fig. 2), the presence of dark coloration^8^ can have similar developmental biological background there as well. Clearly, further studies are needed, preferentially at the genomic level, to recover the underlying mechanism behind the occurrence of rare dark and pale forms in both the eastern sakers (i.e., the ‘Altai form’) and gyrfalcons.

### Conservation implications

Both studied saker populations—as a single species—are listed as endangered^7^, and their basic conservation genetic characteristics (e.g. genetic diversity, inbreeding coefficient) can influence their long-term existence^64^ as well as targets of nature conservation management^65^. Here, we reported (Fig. 7) these values for the two studied saker populations based on 17,095 unlinked SNPs derived from our RADseq dataset^66^. Several questions arise when defining these values based on genome-wide SNPs^67–69^, therefore we may interpret these results with caution. The absolute value of genetic diversity (H_exp_) was relatively high in both populations as it can theoretically only take up values between 0 and 0.5^69^, which is reassuring for conservation work: both populations harbor a substantial genetic diversity which is the basis for their long-term adaptability^64^. In this respect, it is remarkable also that the Pannonian population possesses an apparently higher genetic diversity, although it has undergone a documented bottleneck in the past 80 yr^70^. On the other hand, the studied Tuvan population has also undergone a dramatic population size reduction and has just recently started to recover as a result of conservation actions^17,32,71,72^. Reduction in population size of sakers on the western part of the area has been strongly connected to habitat loss, electrocution and direct persecution, and partly to the steep decline of prey populations, particularly ground-squirrels (genus *Spermophilus*)^73^. Fortunately, the decline of souslik population has recently halted in Hungary^70^ and the Czech Republic^74^ thanks to legal protection, habitat management practices^75^ and re-introductions. Both saker populations studied by us are recovering now and reporting considerable genetic diversity in both populations is promising for the future. Similarly, the inbreeding coefficient is also close to zero (Fig. 7), which can be interpreted as a sign of low levels of inbreeding, although the use of genomic SNPs are even more problematic for assessing this conservation genetic measure^67,68^.

In contrast to some previous findings, our phylogeographic results (Figs. 1, 2, 3, 4, 5 and 6) point to a considerably high level of isolation between saker populations in the Palearctic region. Although we need to admit the limited insight into the overall phylogeographic picture due to lack of samples from the intermediate and adjacent territories (i.e., Middle Asia and the Tibet-Qinghai Plateau). This sets the direction for the continuation of the work. However, we can conclude based on recent results that if a similarly strong isolation exists between all populations, it may also indicate local adaptation^76,77^. If so, the unthoughtful release of captive-bred hybrids or confiscated birds of unknown origin can jeopardize this genetic structure^7^. If eastern and western sakers are adapted to local environmental conditions, their inter-population crossbreeds can bring a serious genetic instability to natural populations via outbreeding depression^65,78^, if released to nature above a genetically critical number. Consequently, if we consider the recent and planned release programs on the eastern range^32^, as well as the often unclear origin of released individuals, the question of anthropogenic hybridization of saker falcons of different geographical origins, as well as their potential hybridization with other closely related species (e.g. gyrfalcon, lanner *F. biarmicus*, and laggar falcon *F. jugger*), are highly relevant conservation issues. Clearly, a range-wide phylogeographic work is needed to better understand the possible adaptive role of population isolation within sakers, because this can be the key to plan genetic rescue^65^ of this globally endangered species. The phylogenetic resolution provided here by RADseq together with its capability to sample the adaptive variance^78^ makes this marker system appealing for this work.

## Material and methods

### Samples and DNA-extraction

To represent the two main groups of sakers (i.e., western and eastern), samples of growing feather from chicks were collected from two populations: the Pannonian population^70^, representing the western group, was sampled for seven birds all across Hungary (samples abbreviated in the analyses as ‘Pann’), and the Tuva population^71^, representing the eastern group, was sampled for ten birds in the Republic of Tuva, Russian Federation (samples abbreviated in the analyses as ‘Tuva’) (Table 1). Sampling of chicks were carried out by plucking one growing feather from the armpit under the permissions of the respective authorities (Hungary: OKTF-KP/56-25/2015; Russian Federation: Federal Supervisory Natural Resources Management Service administrative license №05 from May 10, 2018) using methods and protocols approved by these authorities. Birds with a typical morphology of the Altai falcon were also included from captivity as this form has virtually extinct in the wild: one bird (‘Tuva46’) from ‘Vitasphera’ falconry (Moscow, Russia), and one bird (‘Tuva12’) from ‘Altai falcon’ falconry (Barnaul, Russia) were sampled for this study. These captive birds were descendants of several wild females of an Altai phenotype legally caught in the Altai-Sayan region in 1990s for captive breeding^32^. We supplemented the samples of Altai falcons with a sample (‘Tuva20’) which is an offspring of a mother displaying an Altai phenotype that nested in natural conditions with an eastern saker male in Tuva. Four samples of gyrfalcon, collected from birds of Far Eastern arctic Russia natural population origin confiscated by the All-Russian Research Institute of Environmental Protection, were included from natural populations of Far Eastern arctic Russia (abbreviated as ‘Gyr’) as outgroup samples (Table 1). The work was conducted in accordance with ARRIVE guidelines (https://arriveguidelines.org). In addition, all methods were performed in accordance with the relevant guidelines and regulations. We also note anesthesia was not used and no animals were sacrificed during the study.

Hungarian samples were processed at the laboratory of the Evolutionary Genomics Research Group, Debrecen, whereas the Russian samples were processed at the Core Centrum of the Institute of Developmental Biology Russian Academy of Sciences, Moscow, using exactly the same protocol. DNA was extracted from ethanol (in case of Russian samples) or silica-gel (in case of Hungarian samples) fixed growing feathers by removing the mesenchymal pulp with the blood clots and tips from the calamus^79^. This tissue part was used in isolation of total genomic DNA using a standard mammalian nucleic acid extraction protocol. In short, we incubated the dissected material from samples individually in the extraction buffer (200 mM NaCl, 100 mM Tris–HCl, 5 mM EDTA, 0.2% SDS) with Proteinase K (Thermo Scientific, Waltham, MA, U.S.A.) at 55 °C overnight for complete lysis. Lysed samples were incubated with RNase A (Thermo Scientific) for 30 min at 37 °C. RNase treated samples were incubated in the freezer for 10–15 min with 0.5 V 7.5 M ammonium acetate, then centrifuged with 1 V of chloroform and isoamyl-alcohol (24:1) added. DNA precipitation was performed by adding 1 V ice-cold isopropanol, incubating for 1 h at − 20 °C and centrifugation at maximum speed. After washing with 70% ethanol, the pellets were dissolved and preserved in 10 mM Tris–HCl (pH 8.5). Double-stranded genomic DNA quantity was checked by a Qubit v.3.0 fluorometer (Thermo Scientific), with dsDNA High Sensitivity (HS) Assay Kit, and the integrity of DNA was checked using 1% agarose gel electrophoresis.

### RADseq library preparation and SNP calling

In order to overcome the limited phylogenetic resolution, commonplace with conventional genetic markers within this species, we used RADseq using one type-II restriction enzyme^34^ deployed in the Hungarian lab. To multiplex our samples, we used a system of double index barcoding combined with inline barcodes in the adaptor sequences ligated to the sticky ends: seven different, custom-modified P1 adaptors and three different custom-modified P2 adaptors were used. We used 210 ng of high-quality (i.e., high purity and integrity) genomic DNA (gDNA) from each sample. In presence of the draft genome of *Falco cherrug* ^28^, we predicted the number of expected RAD-loci if using the enzyme PstI and SbfI by in silico digestion as implemented in fragmatic^80^. Based on these results, we selected SbfI-HF (New England Biolabs, Ipswich, MA, U.S.A.) to restriction digest our samples according to manufacturer’s protocol. Modified P1 adaptors were then ligated onto each digested sample, which were subsequently sonicated using a Bioraptor Pico sonicator (Diagenode SA., Seraing, Belgium) for five cycles of ultrasonic shearing of 30 s ‘on’ and 30 s ‘off’. Than DNA fragments came through size selection on both sides using the SPRI Select Beads Kit (Beckman Coulter Inc., Brea, CA, U.S.A.). After blunting and poly-A ligation P2 adaptors were ligated onto the randomly sheared end of our sample DNA using T4 DNA ligase (New England Biolabs). The sub-libraries were pooled equimolarly and went through two-side size selection using the SPRI beads. Compared to the original protocol, a modest PCR amplification was carried out with High-Fidelity PCR Master Mix with HF Buffer (New England Biolabs) in eight separate 50 μl volumes each with 14 amplification cycles. PCR products aliquots were combined before a final size selection on both sides using SPRI beads. After size selection, the library was checked on a Bioanalyzer device (Agilent Technologies Inc, Santa Clara, CA, U.S.A.). The final library was sequenced on an Illumina HiSeq platform providing paired-end, 150 bp long reads at Novogene Co. Ltd. (Beijing, China).

The Illumina reads were de-multiplexed by the process_radtags module of the software package Stacks v.2.2^81^. Further quality filtering and adapter removal were accomplished by using trimmomatic v.0.39^82^ using default values and specifying the custom adapter and primer sequences used during the library preparation as a collection of potential adapter sequences. The filtered reads were aligned to the available draft reference genome assembly (GCF_000337975.1) of saker falcon^28^ using BWA v.0.7.12 with default parameters. The mapped reads were processed with the ref_map module of Stacks, and SNPs were exported from the mapped RAD-loci using the populations module of the software package Stacks. This module was run two-times: (1) with default settings to export all SNPs from the whole dataset (dataset: “ref”); (2) again to export a single random SNP from each RAD-locus (dataset: “rand”) to minimize linkage between the filtered SNPs. The first two datasets were further filtered using vcftools v.0.1.16^83^: “ref” dataset was filtered for maximum 20% individual missingness and minor alleles present in more than two individuals; the dataset “rand” was filtered for maximum 10% individual missingness, minor alleles present in more than two individuals, and sites that are in Hardy–Weinberg-equilibrium at *p* = 0.01 level of significance. The latter dataset was also used to estimate the genetic differentiation between the studied populations by vcftools.

The explicitly root our phylogenetic tree, we mined our RAD loci’s homologous genomic bits from the publicly available reference genome of *F. peregrinus* (assembly: GCA_001887755) and *F. biarmicus* (assembly: GCA_023638135). The RAD loci sequences with an observed heterozigosity of < 0.05 and zero missingness (i.e., present in all individuals) were recovered with Stacks, then checked for polymorphic sites. Loci containing at least one polymorphic site (n = 422) were subject to consensus sequence generation using the EMBOSS tool consambig^84^. Loci sequences were matched against the reference genome of *F. peregrinus* and *F. biarmicus* using blastn v. 2.5.0^85^ with an expected e-value of 1e-25. The genomic region with the highest score was retained and aligned with RAD loci sequences using MUSCLE v. 3.8^86^. Alignments of loci were concatenated with AMAS.py^87^. The resulting fasta file were used in downstream analyses of this dataset.

### Phylogenomic analyses

The whole SNP set (dataset: “ref”) derived from 21 birds was subject to different phylogenetic analyses. First, we used Kimura-2p genetic distance to build a Neighbour-Net phylogenetic network in SplitsTree v.4.14.4^37^ that helped us to locate the samples in the phylogenetic space. Then, we employed a phylogenetic tree reconstruction method to gain insight into the phylogenetic relationship of our samples: (1) we used concatenation to build a maximum likelihood (ML) tree using IQ-tree v.1.6.12^88^ with automatic model selection using ASC and assessing branch robustness via Approximate Likelihood-Ratio Test (aLRT)^89^ and non-parametric bootstrap (bs)^90^. Branches were considered to be statistically supported if aLRT ≥ 80% and bs ≥ 75%.

The phylogenetic analyses using two explicit outgroups, *F. peregrinus* and *F. biarmicus*, utilized to approaches: a maximum parsimony (MP) analysis with 1000 non-parametric bootstrap to test for statistical robustness as implemented in Paup v.4.0a169^91^. We run the heuristic search using 1000 random starting replicates and keeping 10 trees at each branch-swapping step. The resulting trees were saved as phylogram rooted on the sample of *F. peregrinus*. After assuring the same most parsimonious trees were found in 1000 replicates and single search mode, 1000 non-parametric bootstrap pseudo-replicates were run in non-replicated tree search. One tree of the resulting most-parsimonious trees were chosen that reflected the topology of the tree resulting from the maximum likelihood (ML) tree search (see below), and statistical robustness was indicated by superimposing the bs values at the corresponding branch. An additional phylogenetic tree reconstruction using the ML search criterion was conducted in IQ-tree using automatic model selection using the partitioned dataset (without merging data partitions sharing the same evolutionary model) generated from the outgroup dataset. Branch robustness in this analysis was assessed by 1000 aLRT and 1000 ultra-fast bootstrap (UFboot) analyses supplemented with 100 non-parametric bs pseudo-replicates. These values were then superimposed onto the phylogenetic tree at the corresponding branch presented on Fig. 3.

### Population genomic analyses

The unlinked SNP set (dataset: “rand”) derived from 21 birds was used in these analyses all accomplished in R^92^ using always default settings. The filtered vcf file was imported into R by vcfR^93^ and checked the quality of SNPs in each sample by calculating distribution characteristics of read depth and genotype quality as implemented in the package adegenet v.1.3^94^. The latter software was used to carry out PCA and DAPC, whereas we used the package LEA v.2.4.0^95^ for the co-ancestry based snmf calculations. In order to assess the optimal number of PCA axes for DAPC, we used the alpha-score metric to tradeoff between power of discrimination and over-fitting. Finally, we used hierfstat v. 0.04-22^96^ to calculate the basic conservation genetic measures, which were visualized using ggplot2 v.3.3.2^97^. The same software was used when calculating population genetic differentiation.

### Mitochondrial data and haplotype assignment

In order to place our results into a larger context, we sequenced the mitochondrial control region used by Nittinger et al. in their phylogeographic work. We focused only on the ‘eastern’ birds as it is already known that the western sakers possess mitochondrial haplotype only from the western (‘B’) haplogroup. We amplified fragments of the control region sequences using three primer pairs (Table 2). PCR was performed in 20-μl reaction volume containing 1 × buffer solution, 0.2 mM of each dNTP, 0.5 μM of each primer, 2 U of HS Taq polymerase (Evrogen, Moscow, Russian Federation) and 1 μl unquantified DNA extract. Amplification was carried out with a Veriti 96-Well Fast Thermal Cycler (Thermo Scientific) using the following cycling conditions: 5 min denaturation at 95 °C, followed by at 95 °C for 30 s, 1 min annealing at 57–60 °C (Table 2), 1 min 30 s extension at 72 °C for 35 cycles, and 5 min final extension at 72 °C. Contamination controls were included in DNA extractions as well as PCR experiments.

**Table 2:** Oligonucleotide primers used for the amplification of the mitochondrial control region.

primer name	Forward sequence (5′–3′)	Reverse sequence (5′–3′)	Annealing temperature
FCD3	ACTAAACCCATGCCCTGTAT	GAACCAACCGCCCCAAAAAG	58 °C
FCD4	GCCCTTCTCCGAGCCATCTG	GGGTAGGGGGTTTTAAGTTTTTGT	57 °C
FCD5	CGGTTTGCGTATTTGGAGTCA	TCGGGCGGTTTAGGTTTATTGG	60 °C

The PCR products were examined by gel electrophoresis on 2% agarose gel and were directly sequenced in both directions with ABI 3500 sequencer (Thermo Scientific) using the BigDye Termination Cycle Sequencing Kit version 3.1 (Thermo Scientific) following the manufacture’s instruction. Sequences were manually folded by SeqMan (DNASTAR Inc., Maddison, WI, U.S.A.). The sequences were checked for their possible “nuclear copies of mitochondrial loci” (NUMT) origin^98^ using the on-line version of blastn at the National Center for Biotechnology Information (NCBI). Finally, our sequences were aligned into the dataset published by Nittinger et al.^26^ available in GenBank by eye using BioEdit v.7.2.5^99^. We adapted the nomenclature of haplotypes and haplotype groups of the above work. Our sequences were assigned to the published haplotypes by including them in parsimony-based haplotype network building using TCS v.1.21^100^ with default parameters. The resulting network was visually improved using the web-based version of tcsBU^101^.

### Test of introgression

Two datasets were used to test for significant deviation from an allelic distribution resulting from random allele sorting. The dataset generated with the inclusion of an explicit outgroup, *Falco biarmicus*, was subject to classical ABBA/BABA-test (Patterson’s D-statistics) as implemented in the function CalcPopD of the evobiR v 1.3^102^ R package. Furthermore, the *f*_*3*_-statistics was also assessed for the three focal populations using the 3-Population Test as implemented in AdmixTools v. 7.0.2^40^. In order to assess the direction of gene flow, we conducted another analysis in sliding windows as implemented in the script ABBABABAwindows.py from the collection ‘genomics general’ (available at https://github.com/simonhmartin/genomics_general) with a focus on the *F*_*dM*_ statistics^39^ that assesses introgression in lack of an outgroup. The sliding windows size was set to use around 100 sites per window in two arrangements: (1) P1: gyrfalcons, P2: Tuvan sakers, P3: Pannonian sakers; (2) P1: gyrfalcons, P2: Pannonian sakers, P3: Tuvan sakers; (3) P1: Tuvan sakers, P2: Pannonian sakers, P3: gyrfalcons. As the *F*_*dM*_ statistics is positive when there is gene flow between P2 and P3 and negative when there is gene flow between P1 and P3, the visualization of *F*_*dM*_ values along the scaffolds inform us about the direction of gene flow.

### Ethics declarations

Handling of wild birds for sampling and actual sampling was conducted under the permissions of the respective authorities (Hungary: OKTF-KP/56-25/2015; Russian Federation: Federal Supervisory Natural Resources Management Service administrative license №05 from May 10, 2018) using protocols and methods approved by the respective authorities (Hungary: Governmental Office of the Hungarian State; Russian Federation: Federal Supervisory Natural Resources Management Service). The issuances of the above permissions includes the pre-requisite to handle wild birds for sampling using methods and protocols in accordance with international ethical standards of use of wilds birds in research (https://birdnet.org/), which was strictly kept during sampling.

### Supplementary Information

Supplementary Information.

## Data Availability

Mapped RADseq reads for this study have been deposited in the NCBI SRA database under the Bioproject accession number PRJNA819092. Mitochondrial DNA sequences generated for this study have been deposited in NCBI’s Nucleotide collection under the accession numbers: OM937743–OM937753. The fasta file generated by aligning the mined RAD loci from the *Falco peregrinus* and *F. biarmicus* whole genome to the variable RAD loci in our RADseq analysis is available at the data repository of the University of Debrecen (https://doi.org/10.48428/ADATTAR/UUXM4D).
