# Supplementary material for: Phylogenomic insights into the polyphyletic nature of Altai falcons within eastern sakers (Falco cherrug) and the origins of gyrfalcons (Falco rusticolus)
Source: Sci Rep. 2023 Oct 18;13:17800. doi: 10.1038/s41598-023-44534-4 (PMC10584951; doi:10.1038/s41598-023-44534-4)
Supplement: Supplementary file 1 — Supplementary Information. [file 41598_2023_44534_MOESM1_ESM.pdf]

Supplementary Material for Zinevich et al.: Phylogenomic insights into the polyphyletic nature of Altai falcons within eastern Sakers (*Falco cherrug*) and the origins of gyrfalcons (*Falco rusticolus*)

Genotyping statistics (Supp.Fig.1–3.) of the 17,095 filtered SNPs of the 'rand' dataset (i.e., unlinked SNP-set) that was used in population genomic calculations.

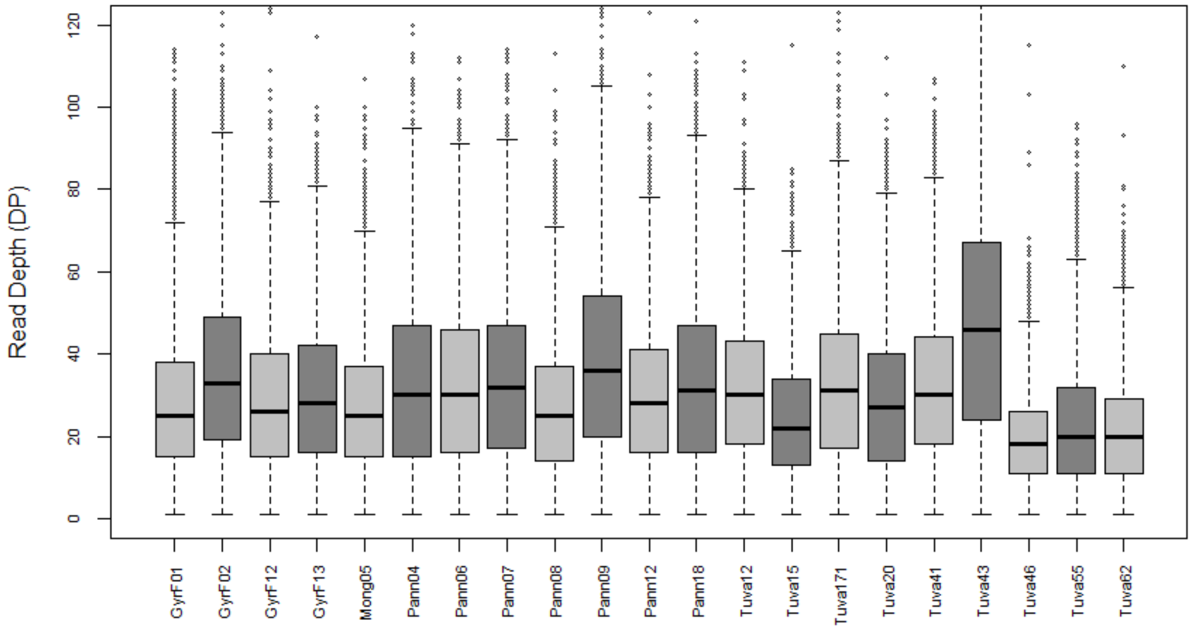

Supplementary Figure 1. Box plot of read depth statistics of the individuals included in the analyses drawn by using the boxplot function of adegraphics v.1.0-12[1].

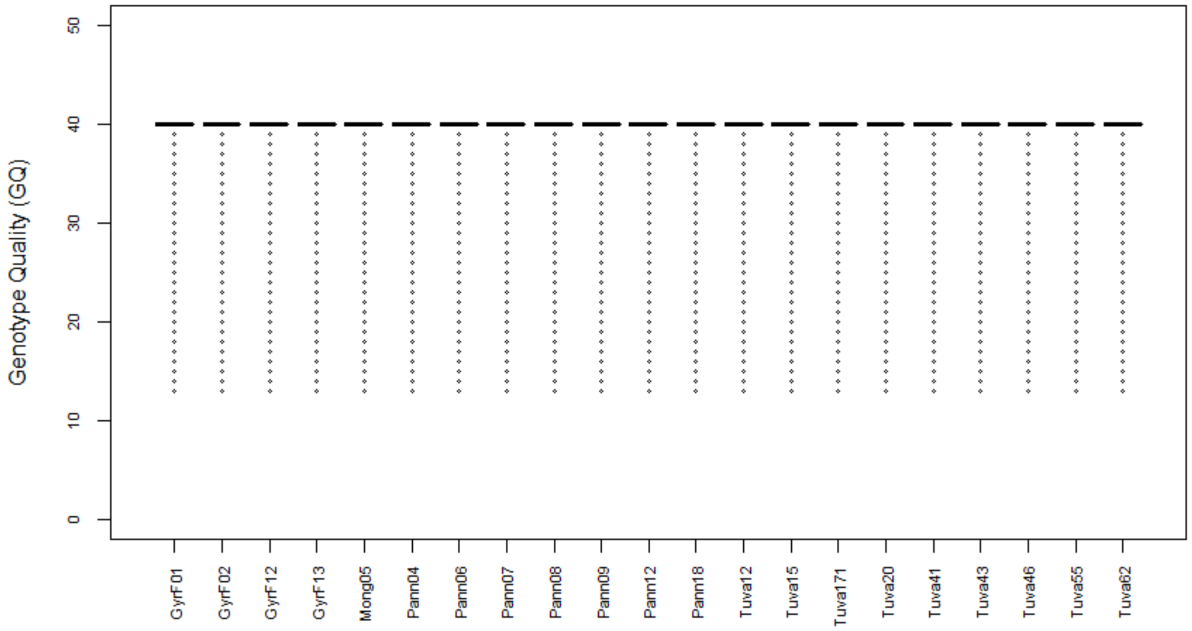

Supplementary Figure 2. Box plot of genotype quality statistics of the individuals included in the analyses drawn by using the boxplot function of adegraphics v.1.0-12[1].

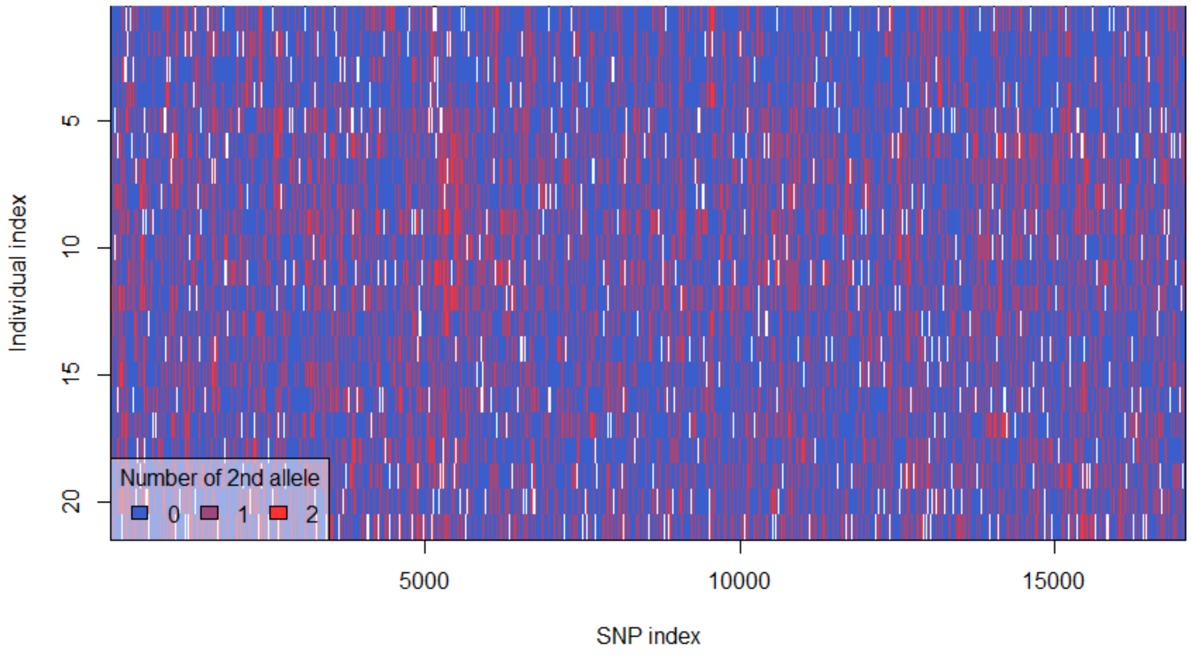

Supplementary Figure 3. Genotype index statistics of the individuals included in the analyses as depicted by the glPlot function of adegenet v.1.3[2].

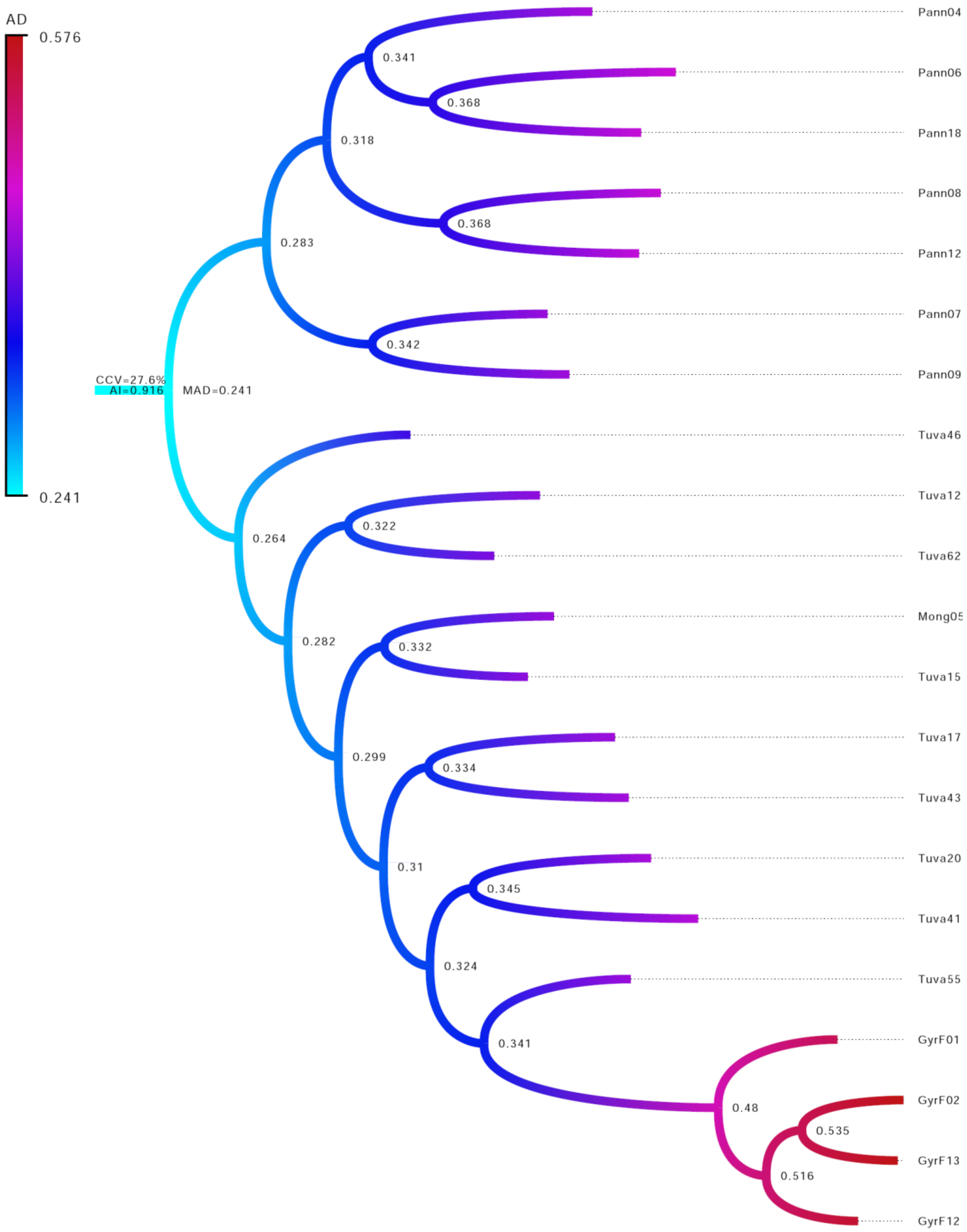

Supplementary Figure 4. Result of phylogenetic rooting based on minimal ancestor deviation as conducted in the software MAD[3]. Figure pasted from the raw output of the analysis software.

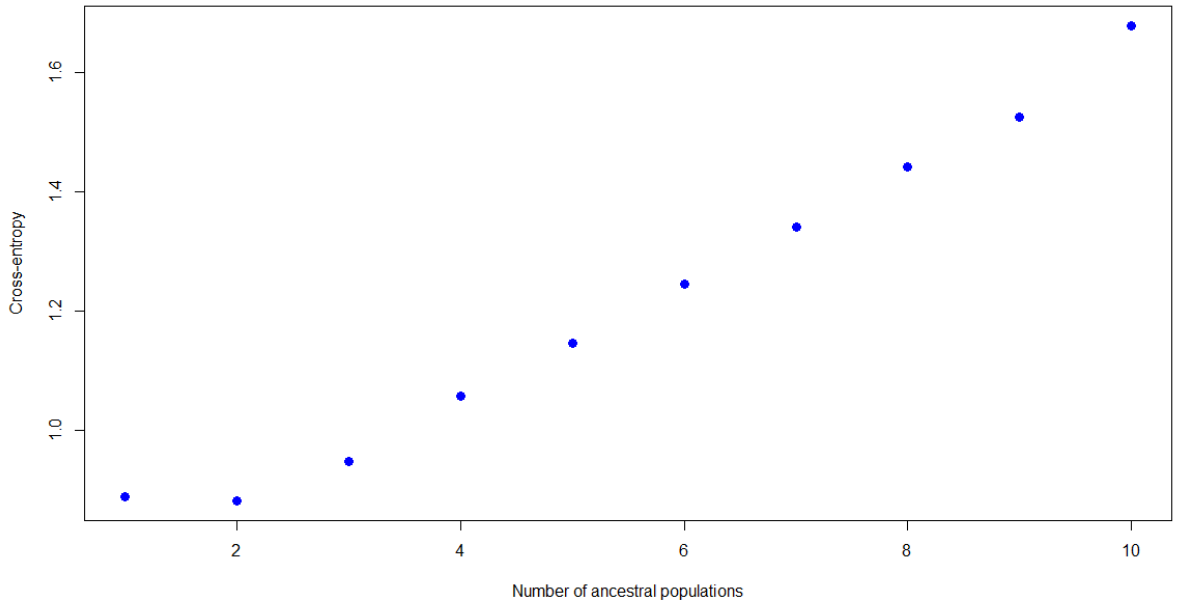

Supplementary Figure 5. Number of potential ancestral populations as assessed by co-ancestry-based sparse non-negative matrix factorization (snmf) analysis of 17,095 unlinked SNP data as implemented in LEA[4].

Supplementary Table 1. Result of f_3-population test, a formal test of admixture in the lack of an exact outgroup sample, with an arrangement of the three studied populations as potential hybrid ('target') population of the source populations ('source1' and 'source2'). Significantly negative values of the f_3 statistic are then evidence of admixture.

**A (source1)**	**B (source2)**	**C (target)**	f_3	**S.E.**	**Z-score**	N_{SNPs}
Pannon	gyrfalcon	Tuva	-0.046762	0.0017	-27.507	17,095
Tuva	gyrfalcon	Pannon	0.099748	0.004071	24.5	17,095
Pannon	Tuva	gyrfalcon	0.209674	0.007446	28.158	17,095

References

1 Siberchicot, A., Julien-Laferrière, A., Dufour, A.-B., Thioulouse, J. & Dray, S. adegraphics: An S4 Lattice-based package for the representation of multivariate data. The R Journal. 9:2. 198–212. https://journal.r-project.org/archive/2017/RJ-2017-042/index.html (2017).

2 Jombart, T. & Ahmed, I. adegenet 1.3-1: new tools for the analysis of genome-wide SNP data. *Bioinformatics* **27**, 3070-3071, doi:10.1093/bioinformatics/btr521 (2011).

3 Tria, F. D. K., Landan, G. & Dagan, T. Phylogenetic rooting using minimal ancestor deviation. Nature Ecology & Evolution 1, 0193, doi:10.1038/s41559-017-0193 (2017).

4 Frichot, E. & François, O. LEA: An R package for landscape and ecological association studies. Methods in Ecology and Evolution 6, 925-929, doi:10.1111/2041-210X.12382 (2015).
